# Comparing fish prey diversity for a critically endangered aquatic mammal in a reserve and the wild using eDNA metabarcoding

**DOI:** 10.1038/s41598-020-73648-2

**Published:** 2020-10-07

**Authors:** Chanjuan Qu, Kathryn A. Stewart, Rute Clemente-Carvalho, Jinsong Zheng, Yuxiang Wang, Cheng Gong, Limin Ma, Jianfu Zhao, Stephen C. Lougheed

**Affiliations:** 1 State Key Laboratory for Pollution Control and Resource Reuse, College of Environmental Science and Engineering, Tongji University, Shanghai, China; 2 Department of Biology, Queen’s University, Kingston, ON Canada; 3 Institute for Biodiversity and Ecosystem Dynamics, University of Amsterdam, Amsterdam, The Netherlands; 4 Key Laboratory of Aquatic Biodiversity and Conservation of Chinese Academy of Sciences, Institute of Hydrobiology of Chinese Academy of Sciences, Wuhan, Hubei China; 5 Administrative Office of Hubei Yangtze Tian’eZhou Baiji National Natural Reserve, Shishou, Hubei China

**Keywords:** High-throughput screening, Biodiversity, Conservation biology

## Abstract

Using environmental DNA (eDNA) metabarcoding, we compared fish diversity in two distinct water bodies within the Yangtze River Basin with known populations of the critically endangered Yangtze finless porpoise (*Neophocaena asiaeorientalis*; YFP*)*: the Tian-e-Zhou Reserve and Poyang Lake. We aimed to create a fish surveying tool for use in the Yangtze River Basin, while also gaining a better understanding of the prey distribution and diversity within two of the remaining strongholds of YFP. 16S rRNA universal primers were developed to amplify fish eDNA. After high-throughput sequencing and stringent data filtering, we identified a total of 75 fish species (6 orders, 9 families, 57 genera) across seasons and regions. Nine of the 75 fish species were among the 28 known YFP prey species, three of which were detected in all water samples. Our eDNA metabarcoding identified many species that had been previously captured using traditional netting practices, but also numerous species not previously collected in these water bodies. Fish diversity was higher in Poyang Lake than in Tian-e-Zhou Reserve, as well as higher in the spring than in summer. These methods provide a broadly applicable tool to quantify fish diversity and distributions throughout the Yangtze River Basin, and to inform conservation strategies of YFP.

## Introduction

Globally, aquatic environments and biodiversity face myriad human-caused threats including pollution, invasive species, overharvesting, diversion, drainage, and climate change^1^. In particular, the 2018 World Wildlife Fund Living Planet Index suggests that freshwater species numbers have declined by 83% on average since 1970, a rate faster than either terrestrial or marine species^2^. Our ability to assess the degree of threat or vulnerability of any species requires that we can reliably detect, quantify, and monitor populations in the wild. Indeed, the conservation of any species requires that we have good data on population demography, distribution and migration patterns, and species ecology. One major cause for the decline of aquatic species of conservation concern is the diminution of prey abundance or changes in their phenology or distribution^1^, precluding the long-term population persistence for many threatened predatory species.

The Yangtze finless porpoise (*Neophocaena asiaeorientalis*; YFP) was once considered a subspecies of the narrow-ridged finless porpoise, but was recognized as a separate species in 2018^3,4^. It inhabits the middle and lower reaches of the Yangtze River, as well as larger tributaries or lakes connected to the river, notably Poyang Lake and Dongting Lake, and is the only freshwater porpoise species in the world^4^. The Yangtze finless porpoise has been listed as Critically Endangered by the IUCN Red List since 2013^5^. The decrease in abundance and diversity of prey of the YFP, particularly fish, is suggested to be a major contributing factor to its decline^6^.

First established in 1992 by the Chinese government for the Yangtze River Dolphin, *Lipotes vexillifer*^7^, the Tian-e-Zhou Baiji National Natural Reserve (hereafter Tian-e-Zhou Reserve) located in Shishou City, Hubei Province, China, is an oxbow of the Yangtze River. It is the main sanctuary for the YFP^8^ containing more than 60 individuals (as of 2015)^9^. The reserve is 21 km long, up to 1–2 km wide, and contains three functional areas: core, buffer and test zones; porpoises are most frequently observed in the core zone, followed by the buffer zone^10^. Importantly, YFP individuals within the Tian-e-Zhou Reserve reproduce and the population increases annually, seeming to thrive without food supplementation^7^. Poyang Lake contains almost half of the extant YFP population (approximately 450 individuals)^11^, but little is known about their feeding habits. Tools to assess the distribution and diversity of prey species would thus provide important insights into the management and recovery of this critically endangered cetacean.

Environmental DNA (eDNA) metabarcoding is a noninvasive and powerful approach for surveying biodiversity and has been applied to numerous taxa including fish^12–15^, other vertebrates^13,16,17^, invertebrates^18^ and zooplankton^19^. It requires only that we collect environmental samples (e.g. water, soil, air or snow), then extract the total DNA contained therein^20^. Environmental DNA is released into environments via species secretions, excretion, shedding, gametes or carcasses^21–23^. After collecting environmental samples and extracting the DNA, researchers can target single gene regions across multiple taxa using conserved primers and obtain data on their presence and relative abundance in a single sample^24^.

Here we apply eDNA metabarcoding to quantify fish diversity in the Tian-e-Zhou Reserve and Poyang Lake, with the goals of (i) creating and testing a tool to survey a broad array of fish diversity in the Yangtze River Basin, and (ii) understanding prey distribution and diversity within two of the remaining strongholds of YFP. By investigating these patterns, we may gain insights on fish communities and seasonal and spatial changes in species richness and relative abundances within the Yangtze River Basin. Quantifying variability in YFP prey fish species will also provide an important tool for managing the habitats of this critically endangered cetacean.

## Materials and methods

### eDNA sampling

**Figure 1:**
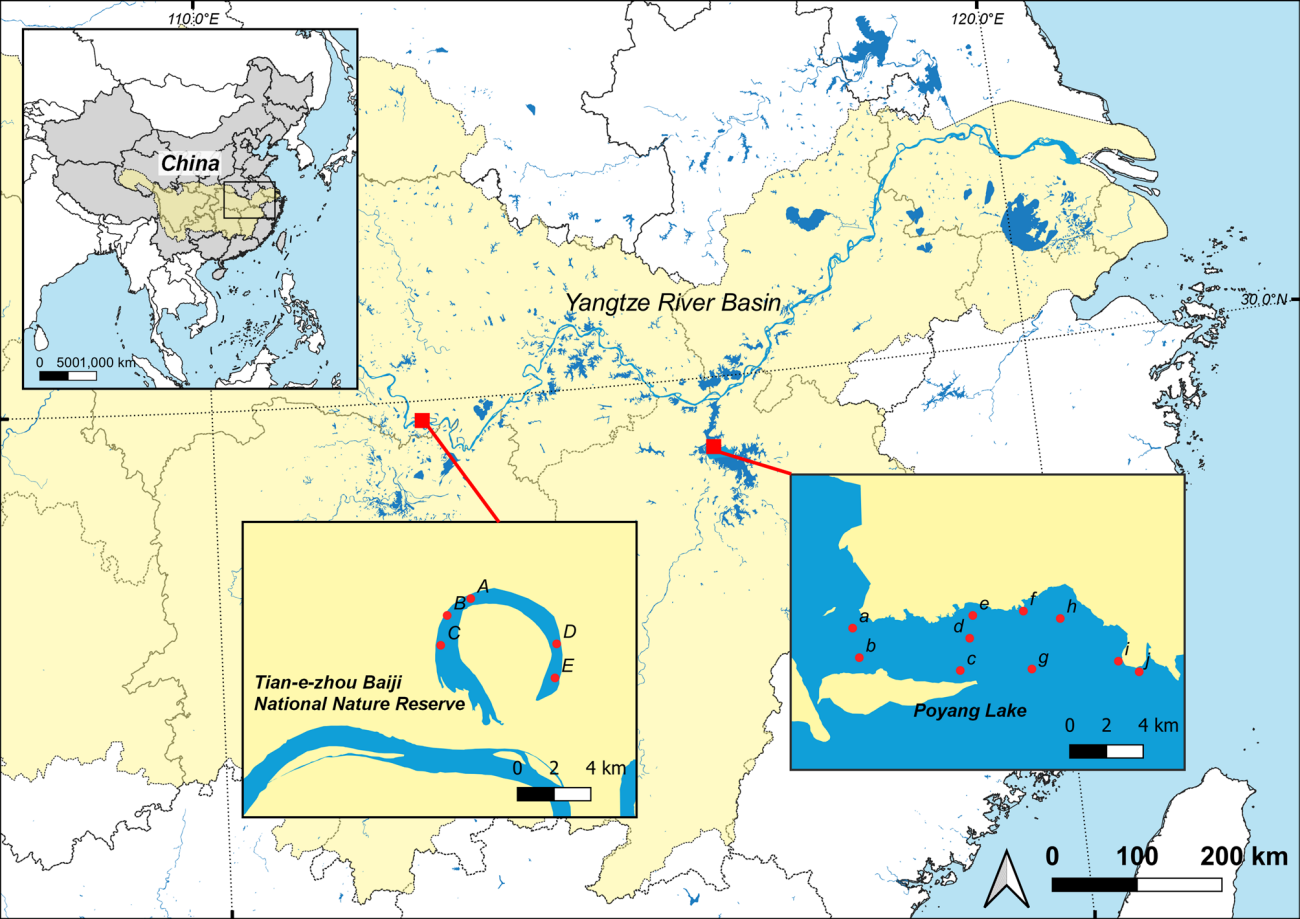
Geographical distributions of 15 sampling sites across 2 regions (insets): Tian-e-Zhou Baiji National Nature Reserve and Poyang Lake. Map created using QGIS version 3.2.0 (QGIS Development Team. 2018. QGIS Geographic Information System. Open Source Geospatial Foundation Project. Retrieved from https://qgis.osgeo.org).

We collected water samples in spring (April 12 to April 18, 2017) and summer (July 3 to July 6, 2017, and July 23 to July 26, 2017), sampling 5 sites in the Tian-e-Zhou Reserve (A through E), and 10 sites in Poyang Lake (a through j) (Fig. 1). Sampling sites spanning the entire length of the Tian-e-Zhou Reserve were identical to those in Stewart et al.^10^. Sites A, B, C and D were located in the core zone (main YFP inhabited area), and site E in the buffer zone (less inhabited area). In Poyang Lake, sampling sites were selected to coincide with locations where local fishermen visually observed YFP frequently. Geographical coordinates for each sampling site were recorded using a handheld GPS unit, and pH, temperature, dissolved oxygen (DO) concentration were measured using portable meters (Supplementary Table S1).

At each sampling site in spring and summer, we used a VanDorn water sampler (GRASP CG-00, GRASP Science and Technology, Beijing, China) to collect 15, 1-L water samples 1 m from the bottom to avoid sediment, with a maximum collection depth of 13 m (Supplementary Table S1). Following standardized protocols to minimize the probability of cross contamination^25,26^ with minor modifications, the sampler was sterilized with 10% diluted bleach between all sampling events, and then rinsed three times with surface water at each site before each sample collection, ensuring that rinse site and actual sampling locale were at least 2–3 m apart. Water samples were filtered using a portable peristaltic pump (Spectra Field-Pro Professional Grade, Spectra Scientific, Toronto, Canada) with 47-mm diameter mixed cellulose-ester filter paper, 0.45 µm pore size^27^. The filter holder (Sterifil Filter Holder, MILLIPORE, Shanghai, China) was also sterilized with bleach and surface water between uses as described above. We used one or two filter papers to filter each 1-L water sample depending on turbidity and clarity of the water. We used 95% ethanol and flame-sterilized tweezers to fold and place the filter paper from filtering each 1-L water sample into a 5 mL Eppendorf tube filled with 95% ethanol. At each sampling site across the two seasons, we also collected a sampling negative control (1 L of distilled water per site that was treated identically to the above water filtering protocol) to test for contamination during the sampling process. All samples were stored in the lab at − 20 °C until eDNA extraction.

### eDNA extraction

Following Thomsen et al.^28^ with minor adjustments to the protocol, each filter was air-dried, rolled up, cut into ca. 1 mm slices and placed in a 2.0 mL lysis tube. 0.3 g of 0.9–2.0 mm stainless steel beads and 740 μL of tissue lysis buffer ATL (QIAGEN) were added to each tube, which were then shaken in an Air Cooling Bullet Blender (Storm BBY24M, NEXT ADVANCE) at high-speed for 3 min. After this, the tubes were incubated at 56 °C for 30 min, followed by another shaking step and incubation step as above. Then 80 µL of 20 mg/mL Proteinase K Solution (BIOLINE) was added to each tube followed by a final incubation at 56 °C for 2 h with agitation. Tubes were then vortexed for 15 s and spun for 5 min (17,000 g). Each sample supernatant (500 μL) was transferred into a new 1.5 mL tube and spun for 1 min (17,000 g).

A volume of 500 µL of chloroform-isoamyl alcohol (24:1)^29–31^ was added to the supernatant of each sample. Each tube was vortexed briefly, shaken for 10 min on a shaker, and spun for 20 min (13,000 rpm). The supernatant (aqueous phase) was then transferred to a new 1.5 mL tube, and this chloroform-isoamyl alcohol step was repeated. 500 µL of isopropanol and 250 µL of 5 M NaCl solution were then added to each tube, which were then vortexed briefly, spun for 1 min (17,000 g), and incubated at − 20 °C overnight. Each tube was then spun for 15 min (15,000 g), and all supernatants were carefully poured off without losing the DNA pellet. 150 µL of 70% ethanol were added to further clean the DNA, with each tube spun for 15 min (15,000 g), and the ethanol carefully decanted. This step was repeated twice. After the residual ethanol was allowed to evaporate, DNA was resuspended by adding 100 µL of elution buffer AE (QIAGEN). Tubes were incubated at 56 °C for 5–10 min, vortexed briefly, and spun for 1 min (15,000 g).

For each sampling site across seasons, three 100 µL DNA extracts from 3 individual 1-L water samples were combined for a final volume of 300 µL (one eDNA sample). All eDNA samples and extracts from sampling negative controls were stored at − 20 °C until PCR. To avoid contamination, we carried out all eDNA extractions in a biosafety cabinet that was irradiated with UV for 30 min prior to processing. All plasticware and pipettes were also UV sterilized between extractions. Before each use, the lab bench was cleaned with freshly-prepared 2% diluted bleach solution.

### Focal prey species

We searched the China Knowledge Network (www.cnki.net) using the term “Yangtze finless porpoise” (in Chinese 江豚) on December 14, 2017 to find articles related to the diet of the finless porpoise. Our final list included 490 papers. From these, we determined that the diet of the YFP was mainly composed of 14 fish species throughout the year (Supplementary Table S2). According to Yang et al.^6^, YFP diet may include an additional 14 fish species (Supplementary Table S2). For development of DNA primers, we thus targeted these 28 fish species, belonging to 4 orders, 8 families and 22 genera.

### Primer design and initial PCR

**Figure 2:**
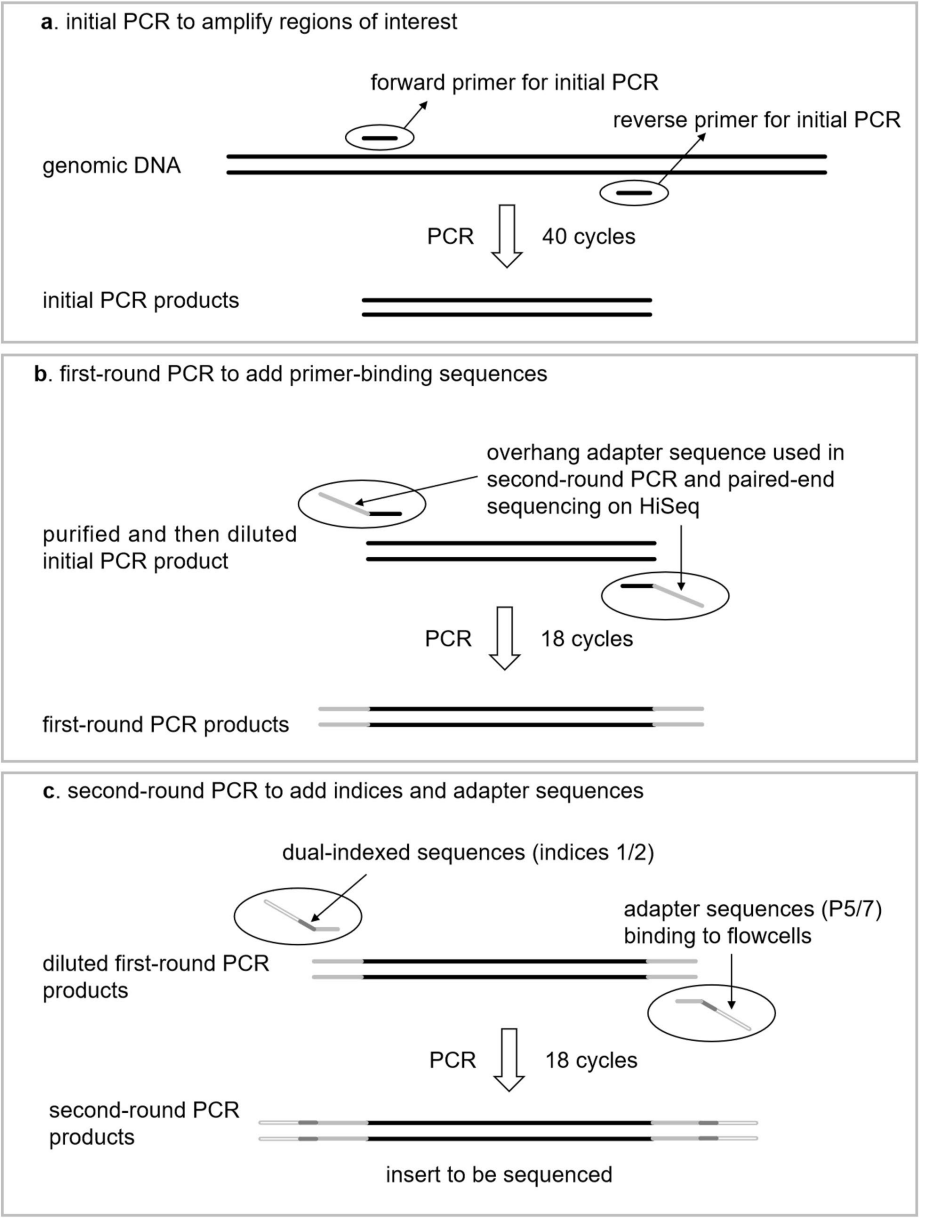
Schematic representation of the (**a**) PCR amplification and (**b** and **c**) paired-end library preparation using a two-step tailed PCR.

We searched the NCBI (National Center for Biotechnology Information; ncbi.nlm.nih.gov) nucleotide database for complete mitochondrial genome sequences of these 28 YFP prey target fish species (Supplementary Table S2). We found a total of 198 16S rRNA sequences that we downloaded and then aligned using ClustalW^32^. We designed a pair of degenerate primers (forward primer, 16S_eDNAF 5′-GTACCTTTTGCATCATGATTYAG-3′ and reverse primer, 16S_eDNAR 5′-TCTYCCCACTCTTTTGC-3′) to amplify a short fragment approximately 133–140 bp in length (Fig. 2) using the BioEdit Sequence Alignment Editor^33^.

We initially tested this primer pair using DNA extracted from tissue samples of 9 fish species (Supplementary Table S2). These fish species were sampled from Poyang Lake in March 2017 using electrofishing licensed by the Duchang Fishery Bureau. All sampling was conducted in accordance with the Regulations of the People’s Republic of China for the Implementation of Wild Aquatic Animal Protection (1993), adhering to all ethical guidelines and legal requirements in China. We also made separate synthetic DNA solutions (Gene Universal, Delaware USA) of prey target sequences for the remaining 19 fish species for which we lacked tissue samples to validate primer specificity (for sequences see Supplementary Table S3). Fragments of the 16S rRNA gene were amplified in 25 μL PCR cocktails containing 12.5 μL of 2 × Taq Mix (including 0.25U/μL Taq DNA polymerase, 2X PCR buffer, 0.4 mM dNTPs, 3.2 mM MgCl_2_ and 0.02% bromophenol blue; Froggabio), 1.2 μL of 10 μM each primer, 0.5 μL of 10 mg/mL Bovine Serum Albumin (BSA; Thermo Fisher Scientific), 3–5 ng of template DNA and nuclease-free water. The PCR cycling conditions were as follows: an initial denaturation step at 95 °C for 5 min followed by 36 cycles of 96 °C for 30 s, 52 °C for 45 s, 72 °C for 45 s and a final extension at 72 °C for 7 min. We performed all PCRs in triplicate. The PCR products were visualized on 1.5% agarose gels stained with RedSafe™ Nucleic Acid Staining Solution. Size standards (100-bp ladder) were included in each gel to verify fragment sizes. Sanger sequencing results (The Centre for Applied Genomics, Toronto, Canada) of amplicons from PCR of DNA extracted from tissue samples and amplicons from PCR of every synthetic DNA solution confirmed the applicability of this primer pair.

For eDNA samples and extracts from field sampling negative controls, we followed the same PCR procedures described above, with the exception that DNA template and nuclease-free water volumes were adjusted to 1.5 μL and 8.1 μL, respectively, and the number of cycles was increased to 40 (Fig. 2). All PCR cocktails included a PCR negative control with nuclease-free water used instead of template DNA. Only when PCR negative controls showed no evidence of a target band did we proceed with the next steps (below). The PCR products of all eDNA samples showed clear target bands of appropriate size (133–140 bp). PCR of all field sampling negative controls showed no evidence of amplification. Because we observed non-target bands from PCR of some eDNA samples, we ran 20 μL of every PCR product from all eDNA samples in a 2% agarose gel and excised the target band manually with single-use scalpel blades. The gel containing the target fragment was purified with the Wizard SV Gel and PCR Clean-Up System (Promega, Madison, WI USA) following the manufacturer’s instructions.

### Library preparation and sequencing

For eDNA samples where we successfully amplified the 16S rRNA fragment of target size, we employed a two-step tailed PCR approach to construct paired-end libraries and to assign unique identification of each sample for multiplexed sequencing (Fig. 2)^34^. The first-round PCR was performed to add primer-binding sequences for subsequent Illumina sequencing. In this step, the primer pairs contained both the degenerate primers above and the primer-binding sequences (Fig. 2; Supplementary Table S4). The 25 μL PCR cocktails contained 12.5 μL of 2 × Taq Mix (Froggabio), 9.25 μL of nuclease-free water, 0.875 μL of 10 μM each primer, 0.5 μL of 10 mg/ml BSA, and 1 μL of 0.1 ng/μL purified initial PCR product. The PCR cycling conditions were as follows: denaturation at 95 °C for 5 min followed by 18 cycles of 95 °C for 30 s, 68 °C for 30 s, 72 °C for 30 s and a final extension at 72 °C for 7 min. The PCR products were subsequently diluted 400 times and used in the second-round PCR as DNA template. The second-round PCR added Illumina adapter sequences and dual indices (combinations of D501-508 and D701-712) to identify different samples for sequencing on the HiSeq platform (Fig. 2; Supplementary Table S4). The 25 μL PCR cocktails contained 12.5 μL of 2 × Taq Mix (Froggabio), 9.26 μL of nuclease-free water, 0.87 μL of 10 μM each primer (Supplementary Table S4), 0.5 μL of 10 mg/mL BSA, and 1 μL of diluted first-round PCR product. The PCR cycling conditions were identical to the first round of PCR.

We used Solid Phase Reversible Immobilisation (SPRI) beads to separately purify 90 (15 sampling sites * 2 seasons * 3 PCR replicates) second-round PCR products, and then used the DeNovix dsDNA Ultra High Sensitivity Evaluation Kit (Wilmington, DE USA) to measure concentrations. Finally, 6 ng of DNA from each purified second-round PCR product were pooled and purified again using SPRI beads. The pooled samples were submitted to the Génome Québec Innovation Centre (Quebec, Canada) for 150 bp, paired-end run on the Illumina HiSeq 4000 platform.

### Bioinformatics and data analysis

Quality control checks were performed on raw read data using FastQC v.0.11.8. No sequences were flagged as being of poor quality. We then used Trimmomatic v.0.32^35^ with “ILLUMINACLIP:HiSeq.adapter.fa:3:30:10:1 MINLEN:122” command under the paired-end mode to remove Illumina adapters^36,37^. After quality filtering and adapter removal, fastq files were converted to fasta files. We used VSEARCH v.2.5.0^38,39^ to dereplicate identical reads (derep_fulllength), and then added the number of identical reads to the header line of the FASTA formatted data file (sizeout). Sequences with < 10 identical reads were removed (minuniquesize 10), reducing the potential for artifacts^40^.

Representative sequences with ≥ 10 identical reads were subjected to a nucleotide (NT) BLAST search (blastn, evalue 0.00001 and perc_identity 99)^41,42^ on NCBI using BLAST + v.2.7.1^43^. The results were imported into MEGAN v.6.15.2^44^, and taxa were assigned using the NCBI-NT Database and default lowest common ancestor (LCA) algorithm with Min Score = 200, Max Expected = 10^−5^, and Min Percent Identity = 99 to discard sequences that fell below our thresholds^39,45^.

We then manually tabulated the sequence abundance of each fish species based on the total number of identical reads for all sequences assigned to each fish species. Similar to Siegenthaler et al.^46^, we considered a species to have been detected if it was present in ≥ 2 PCR replicates. Species detected in only one PCR replicate sample were not considered further, making our results more conservative and less prone to reporting error^39^. We then generated rarefaction curves to assess sequencing coverage per sampling site and season, based on the mean normalized counts of taxonomic units detected with eDNA. A principal coordinates analysis (PCoA) was performed and Jaccard indices were calculated to visualize dissimilarity and similarity among sampling regions and seasons. The Ward’s Hierarchical clustering was used to identify which sites were more similar in species composition. The rarefaction curves, PCoA and clustering analysis were performed using the application ranacapa available at https://gauravsk.shinyapps.io/ranacapa. Bar graphs were generated in RStudio v.1.1.456^47^ with R v.3.5.3^48^ using the package ggplot2 v.3.2.1^49^ to visualize patterns of family and species diversity across sites and seasons. A heatmap was also created in RStudio using the diverse v.0.1.5^50^ and foreign v.0.8–72 packages to visualize the community composition and spatial distribution using presence-absence data for taxonomic datasets.

## Results

### HiSeq sequencing results and initial analysis

The HiSeq paired-end sequencing yielded over 286 million paired-end reads. The read quality scores obtained were similar between libraries, with an average Phred score of 33. We employed stringent filtering parameters (as described above) and retained a high-confidence dataset consisting of ~ 10.1 million reads. The range in number of reads per library (water sample) was between 2168 and 1,413,298 (Supplementary Table S5). Overall, eDNA samples reached sufficient sequencing coverage based on the generated rarefaction curves (Supplementary Fig. S1).

### Total fish species diversity

Initially a total of 101 fish species from 90 libraries was identified, and the taxonomic composition and read numbers were tabulated (Supplementary Table S5). Because these 90 libraries were obtained by using 3 PCR replicates for each of the 30 eDNA samples, and only species present in at least 2 PCR replicates were included in our analyses, we retained 75 fish species for all subsequent analyses, removing 26 fish species detected in only one PCR replicate (Supplementary Tables S6 and S5).

**Figure 3:**
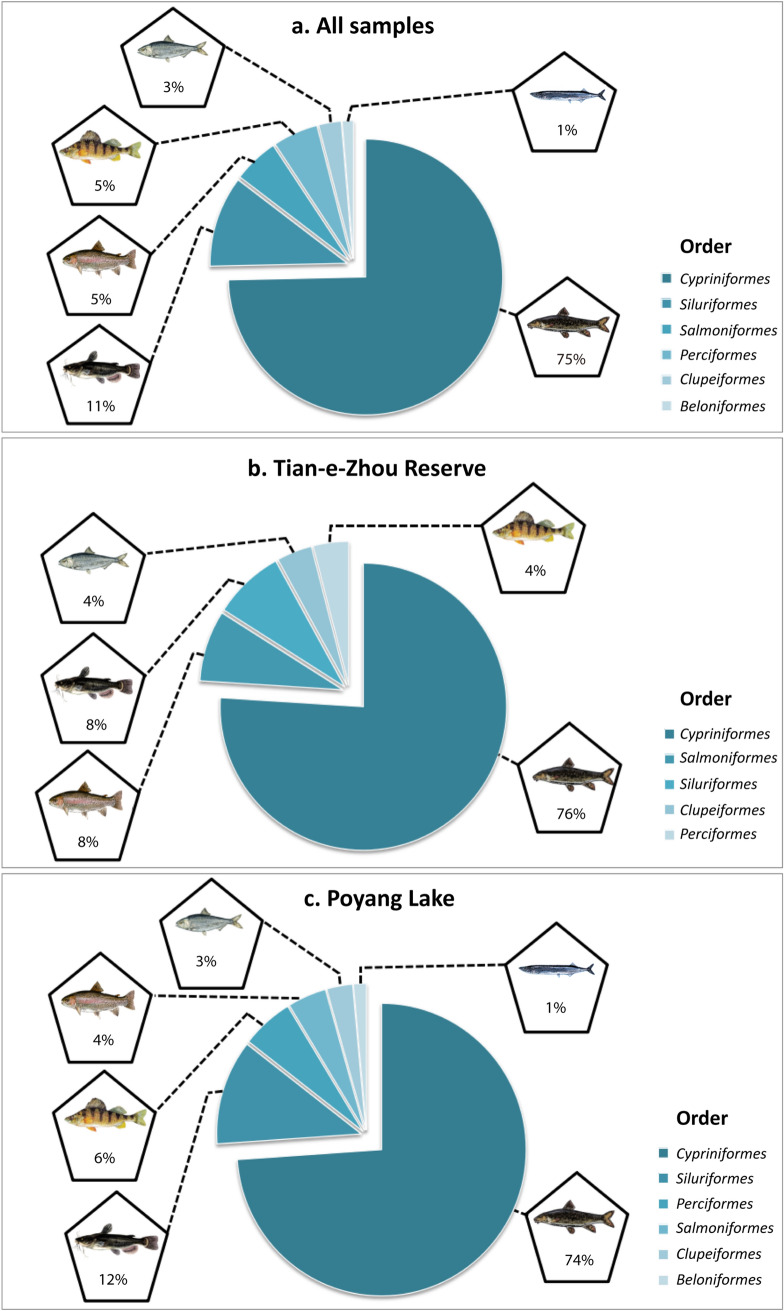
Total proportion of all fish species for each fish order identified (**a**) across seasons and regions, (**b**) within Tian-e-Zhou Reserve, and (**c**) within Poyang Lake.

These 75 fish species belonged to 6 orders, 9 families, and 57 genera (Table 1). At the ordinal level, *Cypriniformes* had the highest diversity, comprising 56 species (75%), followed by 8 species of *Siluriformes* (11%), 4 species of each of *Salmoniformes* (5%) and *Perciformes* (5%), 2 species of *Clupeiformes* (3%), and 1 species of *Beloniformes* (1%) (Fig. 3a). Overall, we detected 4 pelagic species, 53 benthopelagic species (71% of all fish species detected), 17 demersal species, and one *Cyprinidae* hybrid species of unknown ecology; *Dawkinsia tambraparniei* (benthopelagic) had the lowest relative sequence abundance (21 reads), and was only identified in spring samples from Poyang Lake; *Tachysurus nitidus* (demersal) had the highest relative sequence abundance (3,639,140 reads) located in spring and summer samples in both the Tian-e-Zhou Reserve and Poyang Lake (Table 1). Our analysis suggested the presence of a number of non-native fish species including Atlantic salmon (*Salmo salar*), Bull trout (*Salvelinus confluentus*), Largemouth bass (*Micropterus salmoides*), Flathead chub (*Platygobio gracilis*) and Taiwanese salmon (*Oncorhynchus formosanus*), among others.

**Table 1:** Taxonomic composition and total read numbers of 75 fish species from 90 libraries identified in HiSeq analyses of eDNA samples in spring and summer seasons.

Higher classification	Species	English common name (if known)	Habitat	Total	T-spring	T-summer	P-spring	P-summer
Class *Actinopterygii*								
Order *Beloniformes*								
Family *Hemiramphidae*	*Hyporhamphus intermedius*^c^	Asian pencil halfbeak	pelagic	458	0	0	39	419
Order *Clupeiformes*								
Family *Engraulidae*	*Coilia lindmani*^h^	Lindman's grenadier anchovy	pelagic	23,056	0	2015	14,032	7009
	*Coilia mystus*	Osbeck's grenadier anchovy	pelagic	3030	0	465	1215	1350
Order *Cypriniformes*								
Family *Cobitidae*	*Cobitis elongatoides*^f^	–	demersal	38	0	0	38	0
	*Cobitis lutheri*	Luther's spiny loach	benthopelagic	28	0	0	0	28
Family *Cyprinidae*	*Acanthorhodeus chankaensis*^b,c^	Khanka spiny bitterling	benthopelagic	267,561	99,785	3484	115,456	48,836
	*Acheilognathus imberbis*^c^	–	benthopelagic	78,353	47	142	70,284	7880
	*Acheilognathus meridianus*^c^	–	benthopelagic	22	0	0	22	0
	*Acrossocheilus fasciatus*^c^	–	benthopelagic	19,911	1703	218	11,791	6199
	*Acrossocheilus kreyenbergii*^c^	–	benthopelagic	1,338,412	24,489	10,738	653,389	649,796
	*Acrossocheilus parallens*^c^	–	benthopelagic	48,664	13,397	494	20,233	14,540
	*Ancherythroculter kurematsui*^c^	–	benthopelagic	3906	778	140	1250	1738
	*Ancherythroculter lini*	–	benthopelagic	835	231	97	171	336
	*Balantiocheilos melanopterus*^h^	Tricolor sharkminnow	benthopelagic	765	0	0	490	275
	*Barbodes banksi*^h^	–	benthopelagic	14,524	1,809	348	7709	4658
	*Barbodes binotatus*^h^	Spotted barb	benthopelagic	423	121	0	203	99
	*Barbus ciscaucasicus*^g^	Terek barbel	benthopelagic	5747	635	42	3379	1691
	*Carassius auratus*^a,b,c^	Goldfish	benthopelagic	2190	196	0	1435	559
	*Carassius carassius*^f^	Crucian carp	demersal	785	0	0	358	427
	*Chanodichthys erythropterus*^b,^^c^	Predatory carp	benthopelagic	6198	1004	198	3247	1749
	*Cyclocheilichthys janthochir*^h^	–	benthopelagic	98	0	0	0	98
	*Cyprinidae* hybrid sp*.*	–	*unknown*	758	84	0	464	210
	*Cyprinus megalophthalmus*	–	benthopelagic	1727	219	0	1032	476
	*Dawkinsia tambraparniei*^i^	–	benthopelagic	21	0	0	21	0
	*Discherodontus ashmeadi*^h^	–	benthopelagic	828	77	0	482	269
	*Elopichthys bambusa*^b,c^	Yellowcheek	benthopelagic	77,901	47,393	12,981	108	17,419
	*Eremichthys acros*^e^	Desert dace	demersal	102	0	0	102	0
	*Gnathopogon strigatus*	Manchurian gudgeon	benthopelagic	1857	0	0	1664	193
	*Gobiobotia macrocephala*^j^	–	benthopelagic	954,296	2064	889	768,424	182,919
	*Hemibarbus labeo*	Barbel steed	benthopelagic	188	188	0	0	0
	*Hemibarbus umbrifer*	–	benthopelagic	118	118	0	0	0
	*Hemiculter leucisculus*^a,b,c^	Sharpbelly	benthopelagic	6321	2387	500	1309	2125
	*Hemiculterella sauvagei*	–	benthopelagic	118	70	48	0	0
	*Hypophthalmichthys molitrix*^a,b,c^	Silver carp	benthopelagic	7360	3098	3356	274	632
	*Margariscus margarita*^e^	Allegheny pearl dace	demersal	9286	59	74	5207	3946
	*Onychostoma barbatulum*^c^	Taiwan shoveljaw carp	benthopelagic	7030	818	114	4055	2043
	*Osteobrama cotio*^i^	–	benthopelagic	7883	556	0	4598	2729
	*Platygobio gracilis*^e^	Flathead chub	demersal	47	0	0	47	0
	*Procypris rabaudi*	Rock carp	benthopelagic	22	0	0	22	0
	*Pseudaspius leptocephalus*	Redfin	benthopelagic	22	0	0	0	22
	*Pseudohemiculter dispar*^c^	–	benthopelagic	1307	278	62	228	739
	*Pseudolaubuca engraulis*^a,b,c^	–	benthopelagic	943,566	235,527	59,333	269,509	379,197
	*Ptychocheilus umpquae*^e^	Umpqua pikeminnow	benthopelagic	752	0	0	752	0
	*Puntioplites waandersi*	–	demersal	1365	125	0	895	345
	*Rutilus rutilus*^f^	Roach	benthopelagic	256	0	0	256	0
	*Sarcocheilichthys lacustris*	–	benthopelagic	79	0	0	79	0
	*Sarcocheilichthys. sinensis*^c^	Chinese lake gudgeon	benthopelagic	8810	0	0	8810	0
	*Saurogobio immaculatus*	–	benthopelagic	1800	0	0	775	1025
	*Schizothorax plagiostomus*	–	benthopelagic	3494	396	0	2084	1014
	*Semotilus atromaculatus*^e^	Creek chub	demersal	56,913	1,393	334	52,909	2277
	*Sinibrama macrops*^c^	–	benthopelagic	2642	711	443	480	1008
	*Spinibarbus denticulatus*^c^	–	benthopelagic	4364	595	24	2294	1451
	*Squalidus argentatus*^a,b,c^	–	benthopelagic	401,722	139,883	20,365	123,313	118,161
	*Squalidus multimaculatus*^j^	–	pelagic	2521	389	0	1904	228
	*Squalidus wolterstorffi*	–	benthopelagic	67	67	0	0	0
	*Squalius lepidus*^g^	–	benthopelagic	119	0	0	32	87
	*Tinca tinca*^c^	Tench	demersal	2275	685	981	400	209
	*Toxabramis houdemeri*	–	benthopelagic	153	100	0	0	53
	*Xenocypris davidi*^a,b^	–	benthopelagic	334	308	26	0	0
Order *Perciformes*								
Family *Centrarchidae*	*Micropterus salmoides*^c,^^e^	Largemouth black bass	benthopelagic	39,971	43	0	31,570	8358
Family *Gobiidae*	*Rhinogobius brunneus*^c^	Amur goby	demersal	25,027	0	0	0	25,027
	*Rhinogobius cliffordpopei*^c^	–	benthopelagic	20,865	0	0	0	20,865
	*Rhinogobius giurinus*^a,b,c^	–	demersal	1,721,934	4030	171,152	196,138	1,350,614
Order *Salmoniformes*								
Family *Salmonidae*	*Oncorhynchus formosanus*^e^	–	demersal	14,513	7736	1820	3533	1424
	*Prosopium cylindraceum*^e^	Round whitefish	benthopelagic	44,498	22,508	6459	9985	5546
	*Salmo salar*^e^	Atlantic salmon	benthopelagic	27,605	7656	1791	6632	11,526
	*Salvelinus confluentus*^e^	Bull trout	benthopelagic	61	61	0	0	0
Order *Siluriformes*								
Family *Bagridae*	*Coreobagrus brevicorpus*^d^	Korean stumpy bullhead	benthopelagic	4417	0	0	4417	0
	*Pelteobagrus eupogon*^c^	–	demersal	11,511	0	0	10,874	637
	*Pelteobagrus intermedius*^c^	–	demersal	15,137	304	145	14,603	85
	*Pseudobagrus ondon*^c^	–	benthopelagic	49,896	176	0	49,627	93
	*Pseudobagrus vachellii*^c^	–	demersal	58,527	213	126	42,411	15,777
	*Tachysurus nitidus*^a,c^	–	demersal	3,639,140	18,730	7998	2,970,666	641,746
Family *Siluridae*	*Silurus asotus*^a,c^	Amur catfish	demersal	25,131	0	0	12,784	12,347
	*Silurus lanzhouensis*	–	demersal	42	0	0	0	42

Species listed are those identified from the BLAST search and NCBI-NT Database based on 99% identity of compared 16S rRNA gene segment. T = Tian-e-Zhou Reserve; P = Poyang Lake.

“Cyprinidae hybrid sp.” represents *Cyprinus carpio* 'mirror' x *Cyprinus carpio 'singuonensis'.*

The Latin name, higher classification, English common name and habitat of fish species are based on https://www.fishbase.se/.

^a^9 finless porpoise prey species.

^b^10 fish species that have been captured using fishing nets in Tian-e-Zhou Reserve.

^c^31 fish species that have been captured using fishing nets in Poyang Lake.

^d^(possible) introduced species outside of its native range.

^e^Non-native*—*North American.

^f^Non-native*—*European.

^g^Non-native*—*Western Asia.

^h^Known from Southeast Asia.

^i^India.

^j^Endemic to South Korea.

### Fish species diversity in different regions

In total, we identified 50 fish species in the Tian-e-Zhou Reserve, and 69 fish species in Poyang Lake (Tables 1 and 2). Of these, 44 species were common across the Tian-e-Zhou Reserve and Poyang Lake samples, which thus shared 59% of the total 75 fish species; 6 species were detected in the Tian-e-Zhou Reserve only, and 25 species were unique to Poyang Lake. We found significantly more fish species in Poyang Lake than in Tian-e-Zhou Reserve samples (2-way ANOVA with season and region as main effects: *F*_(region)_ = 25.53, *p* <  < 0.001).

**Table 2:** Total number of fish species identified across regions (Tian-e-Zhou Reserve and Poyang Lake), sampling sites, and seasons (spring and summer).

Region	Sampling site	Number of fish species						
		Spring	Summer	Spring	Summer	Spring	Summer	Total
Tian-e-Zhou Reserve	A	19	23	48	34	50		75
	B	28	17					
	C	21	23					
	D	20	18					
	E	41	20					
Poyang Lake	a	40	17	62	58	69		
	b	40	36					
	c	36	31					
	d	34	31					
	e	42	39					
	f	39	32					
	g	35	21					
	h	35	36					
	i	37	28					
	j	30	37					

The number from left to right represents the number of fish species identified in spring samples at a sampling site, the number of fish species identified in summer samples at a sampling site, the number of fish species identified in all spring samples in a region, the number of fish species identified in all summer samples in a region, the number of fish species identified in all samples in a region, and the number of fish species identified in all samples, respectively.

The number of taxa clearly varied between the two regions. The fish species identified in the Tian-e-Zhou Reserve belonged to 5 orders, with 38 species (76%) of *Cypriniformes* followed by 4 species each of *Salmoniformes* (8%) and *Siluriformes* (8%), and 2 species each of *Clupeiformes* (4%) and *Perciformes* (4%) (Fig. 3b); the fish species identified in Poyang Lake belonged to 6 orders, with 51 species (74%) of *Cypriniformes*, followed by 8 species of *Siluriformes* (12%), 4 species of *Perciformes* (6%), 3 species of *Salmoniformes* (4%), 2 species of *Clupeiformes* (3%), and 1 species of *Beloniformes* (1%) (Fig. 3c). *Beloniformes* is a new order for the Tian-e-Zhou Reserve. Clearly in terms of species richness *Cypriniformes* dominated in both regions.

### Fish species diversity in different seasons

In the Tian-e-Zhou Reserve, we detected 48 fish species in the spring and 34 fish species in the summer; in Poyang Lake, we detected 62 fish species in the spring and 58 fish species in the summer (Tables 1 and 2). Of these, 30 species were common to spring and summer samples from Tian-e-Zhou Reserve and Poyang Lake; 4 and 11 species were identified only in spring samples from Tian-e-Zhou Reserve and Poyang Lake, respectively, while 6 were identified only in summer samples from Poyang Lake; there were no fish species unique to the summer samples of the Tian-e-Zhou Reserve. For both regions, the detected number of species in the spring was greater than that of summer (2-way ANOVA with season and region as main effects: *F*_(season)_ = 7.15, *p* = 0.013).

Jaccard similarity analysis indicated that species richness between spring and summer samples in Poyang Lake was most similar (0.74), species richness between spring and summer samples in Tian-e-Zhou Reserve was the third most similar (0.64), while species richness between summer samples of Tian-e-Zhou Reserve and spring samples of Poyang Lake were least similar (0.50; see Table 3). Overall, despite these seasonal differences, fish species composition of samples taken from the same water body were more similar than between.

**Table 3:** Jaccard index of fish species between all pairs of region-season combinations.

Sample sets	Tian-e-Zhou Reserve*—*Summer	Poyang Lake*—*Spring	Poyang Lake*—*Summer
Tian-e-Zhou Reserve*—*Spring	0.64	0.59	0.66
Tian-e-Zhou Reserve*—*Summer	–	0.50	0.53
Poyang Lake*—*Spring		–	0.74
Poyang Lake*—*Summer			–

**Figure 4:**
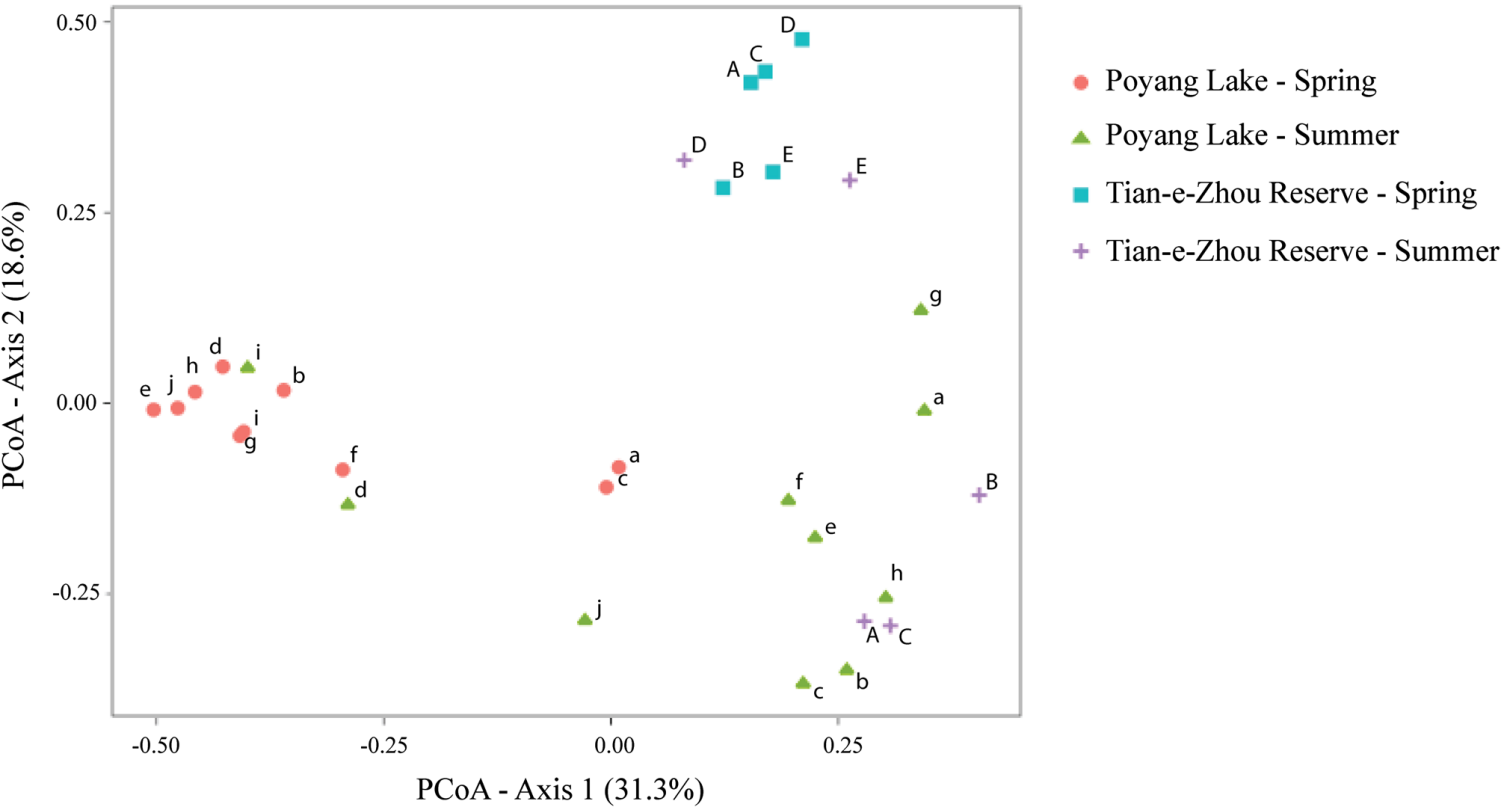
Ordination analysis representing similarity in community composition based on eDNA taxonomic identification using Jaccard index.

**Figure 5:**
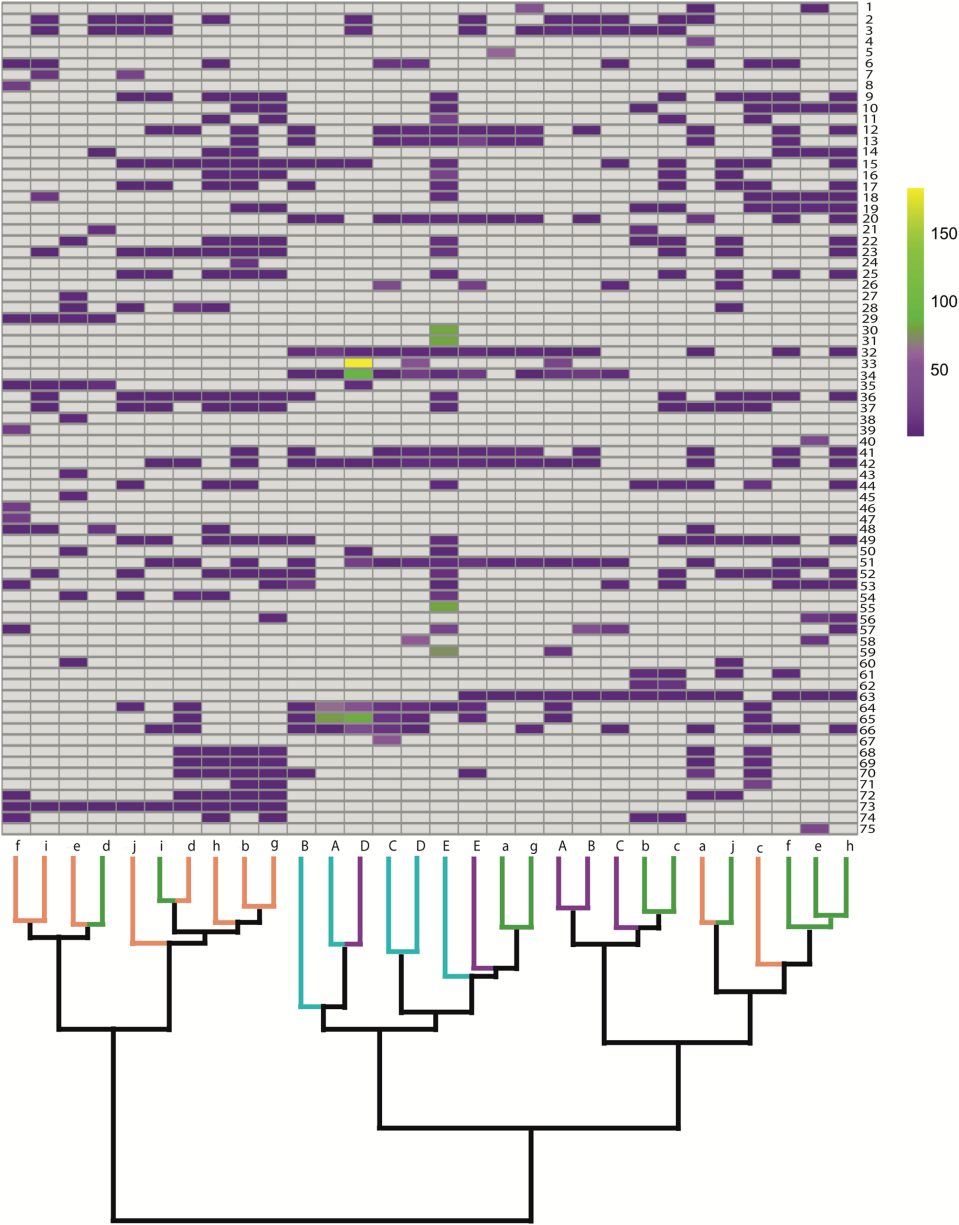
Heatmap and cluster analysis of fish species composition of different samples in the Tian-e-Zhou Reserve and Poyang Lake in the spring and summer. Rows correspond to species IDs (see Table 1 or Supplementary Table S6), columns correspond to each sampling site and season. Bar colors refer to number of reads (normalized), with lighter colors indicating higher values (white represents absence). Cluster plot uses Ward's Hierarchical clustering method; sites with similar taxonomic composition cluster together. Color codes follow Fig. 4 (PCoA).

Plots from our principal-coordinates analysis (PCoA) showed that spring samples from both regions, Tian-e-Zhou Reserve and Poyang Lake, were well separated from all other samples along the first PCoA axis explaining 31.3% of the variation in the data; summer samples were more similar and separated partially along the second PCoA axis explaining 18.6% of the variation in the data set (Fig. 4). This result was concordant with the result of our cluster analysis (Fig. 5).

In the Tian-e-Zhou Reserve, *Micropterus salmoides* and *Pseudolaubuca engraulis* had the lowest and highest relative sequence abundance in spring samples respectively, changing in the summer samples to *Spinibarbus denticulatus* and *Rhinogobius giurinus*; in Poyang Lake, *Dawkinsia tambraparniei* and *Tachysurus nitidus* had the lowest and highest relative sequence abundance in spring samples, respectively, while again these switched to *Pseudaspius leptocephalus* and *Rhinogobius giurinus* in the summer samples (Table 1; Fig. 5). Overall, *Pseudolaubuca engraulis*, *Rhinogobius giurinus*, and *Tachysurus nitidus* had the highest relative sequence abundances.

### Fish species diversity at different sampling sites

We identified different fish species richness in samples from different seasons at different sampling sites within regions. In general, the number of fish species detected in the spring sample was greater than that in the summer sample at each sampling site, except for sampling sites A, C, h, and j (Table 2). Overall, the number of species identified in the sampling sites from Poyang Lake in the spring and summer were higher than that in the sampling sites from Tian-e-Zhou Reserve in spring and summer, respectively. There were a few exceptions. For sampling site E from the Tian-e-Zhou Reserve, the spring sample contained higher richness than spring samples of most sampling sites in Poyang Lake. For sampling sites a and g from Poyang Lake, summer samples had lower richness than that of the summer samples of some sampling sites in Tian-e-Zhou Reserve (Table 2).

Across all 30 eDNA samples (with the exception of Tian-e-Zhou Reserve spring samples at sites A-D), we detected species inhabiting different water depths (pelagic, benthopelagic and demersal; Supplementary Fig. S2). For each sample, the number of benthopelagic fish species was highest, followed by demersal fishes and pelagic species. For the Tian-e-Zhou Reserve, 11 fish species were common to all sampling sites in the spring, and 10 species were common to all sampling sites in summer; in contrast, Poyang Lake had 17 and 6 species common to all sites in spring and summer, respectively (Supplementary Table S7). In total, 4 fish species were found in every sample in our study: *Acrossocheilus kreyenbergii*, *Pseudolaubuca engraulis*, *Rhinogobius giurinus*, and *Tachysurus nitidus*.

The relative abundance of fish species as reflected in sequence reads in the summer samples of the Tian-e-Zhou Reserve was similar to that of the summer samples in Poyang Lake, where most samples contained a similar dominant species, *Rhinogobius giurinus*, in high relative abundance (Supplementary Fig. S3). The dominant species in most spring samples of Tian-e-Zhou Reserve was *Pseudolaubuca engraulis* and was *Tachysurus nitidus* in most spring samples of Poyang Lake. In the Tian-e-Zhou Reserve spring samples, we recorded the highest number of reads for *Cyprinidae*, whereas *Gobiidae* had the highest number of reads in summer; in the spring samples of Poyang Lake, *Bagridae* appeared most abundant, switching to *Cyprinidae* and *Gobiidae* in the summer (Supplementary Fig. S4).

### Prey fish of Yangtze finless porpoise

Of the 75 fish species detected using eDNA metabarcoding, 9 were known YFP prey fish species (Table 1). Three of the 9 prey species detected were common across all 30 eDNA samples among regions and seasons: *Pseudolaubuca engraulis, Rhinogobius giurinus*, and *Tachysurus nitidus*. The two regions generally shared the same YFP prey species, with Tian-e-Zhou Reserve containing one prey species not detected in Poyang Lake, and Poyang Lake similarly containing one prey species not found in the Tian-e-Zhou Reserve.

## Discussion

Environmental DNA metabarcoding is increasingly employed to survey aquatic biodiversity, used successfully in lentic^51^, lotic^15^, estuarine^52^, coastal^42^ and deep-water marine systems^53^. Using this metabarcoding approach, we set out to design DNA primers that would allow us to assess the spatiotemporal dynamics of fish populations and communities across the Yangtze River Basin, including the 28 known prey fish species of the YFP. As would be expected given the geographic extent of our sampling and the taxonomic breadth of the prey species that we used to design our primers, we conservatively found the eDNA signatures of 75 fish species in the two regions that we surveyed across two seasons. Our eDNA investigations revealed differences in species richness between regions and seasons, and suggested that this method would be useful in mapping fish diversity in the Yangtze River and associated water bodies, including both introduced species (Table 1) and prey species of the YFP. Although we sampled from the water column typically one meter from the bottom for both water bodies (Supplementary Table S1), the fish identified with our eDNA metabarcoding approach were not just demersal but included benthopelagic and pelagic species as well.

### Species diversity identified in different regions

Based on the presence of eDNA, we detected 19 more species in Poyang Lake than that in the Tian-e-Zhou Reserve overall. This is perhaps not surprising as Poyang Lake is markedly larger than the Tian-e-Zhou Reserve and is an open system, with inflows from the Ganjiang, Fuhe, Xinjiang, Raohe and Xiuhe Rivers, and connection to the Yangtze River via a channel^54^. In contrast, the Tian-e-Zhou Reserve is effectively isolated from the Yangtze River, only hydrologically linked through sluice gates during episodic flooding. Of the two focal regions in this study, previously published data on fish species that have been captured via traditional net survey methods allow for some comparisons with our eDNA survey data. In the Tian-e-Zhou Reserve, our eDNA surveys detected 10 fish species that had also been caught using fishing nets (Table 1): Gong et al.^55^ conducted net surveys and analysis of the year-round fish resources of the Tian-e-Zhou Reserve, capturing *Carassius auratus*, *Chanodichthys erythropterus*, *Elopichthys bambusa*, *Hemiculter leucisculus*, *Hypophthalmichthys molitrix*, *Rhinogobius giurinus*, *Squalidus argentatus* and *Xenocypris davidi*; additional net surveys by Gong et al.^56^ resulted in capture of another fish species, *Pseudolaubuca engraulis*; *Acanthorhodeus chankaensis* was further detected using net surveys in the Tian-e-zhou Reserve^57^. In Poyang Lake, we detected 31 fish species using eDNA that had also been caught using fishing nets (Table 1): Yang et al.^58^ and Jin et al.^59^ conducted net surveys of fish during spring, summer and autumn seasons, capturing 16 fish species; an additional, four fish species have been reported from Poyang Lake^60–62^ and 11 species in the broader Poyang Lake Basin^54,63^. Clearly future research should focus on the simultaneous comparisons of eDNA and net surveying within the same regions to directly assess sampling labor effort and accuracy; however, the fact that we detected many species reported from traditional net surveys gives us confidence that the patterns that we report here are real.

### Fish species diversity in different seasons and at different sampling sites

We detected greater species richness in spring versus summer samples in both regions and at most of the sampling sites (Table 2). May through July encompasses the breeding season of most fish species in the Yangtze River^64^ and it is thus possible that detection probabilities were higher during our spring sampling simply because there were more eDNA sources for some species (e.g. gametes, fish fry)^23^. Although the species composition among sites in the same region had higher similarity (Table 3), changes in eDNA relative sequence abundance and detection of fish species at different sampling sites (Supplementary Fig. S3) may also reflect shifts in habitat use and distribution^10,65^. For example, Stoeckle et al.^52^ found that variation in fish eDNA detections reflected seasonal presence and habitat preferences determined using traditional surveys in coastal and estuarine marine habitats.

### Prey fish of Yangtze finless porpoises

We found that three YFP prey fish species had among the highest number of total reads of any detected species (*Pseudolaubuca engraulis*—943,566, *Rhinogobius giurinus*—1,721,934 and *Tachysurus nitidus*—3,639,140), implying our metabarcoding approach may provide important insights into prey distributions and potentially relative abundances. Other researchers have found eDNA concentration to be positively related to field-measured density, biomass, or proportion of surveyed transects occupied in amphibians^66^, and fish^67–69^. Evans et al.^13^ further illustrated the potential of eDNA metabarcoding approaches for improving quantification of aquatic species diversity in natural environments and showed how eDNA metabarcoding can serve as an index of macrofaunal species abundance. With further comparisons between metabarcoding sequence read numbers and YFP prey species abundance, future conservation efforts could become more focused on managing or supplementing fish population numbers with hopefully positive consequences for YFP population persistence.

### Sequence cut-offs and other caveats of our methodology

To our knowledge, sequence processing thresholds are not standardized for species diversity assessment using eDNA metabarcoding. For example, Miya et al.^34^ used 97% similarity to identify fish species, Valentini et al.^51^ used 98% similarity to identify amphibians and bony fish species, while Sato et al.^41^, Yamamoto et al.^42^, and Simmons et al.^70^ used 99% similarity to identify fish species. We explored the implications of sequence similarity cut-offs, comparing 97%, 98% and 99% similarity as our thresholds. Unsurprisingly, the results did vary depending on this cut-off with the number of fish operational taxonomic units (OTUs) detected increasing as we increased the threshold from 97 to 99%. Similarly, different authors have used different BLAST E-value (e.g. 10^−5^, 10^−10^ or 10^−20^)^16,34,36^. When we align a sequence to the nucleotide database for species identification, a higher sequence similarity, smaller BLAST E-value, and higher bit-score usually indicate sequence or species matches of higher quality^44^. We ultimately decided to use a 99% similarity cut-off, an E-value of 10^−5^, and set the lowest bit-score to 200. If there were to be internationally recognized values for these parameters for each gene and taxonomic group, the normalization of sequence processing steps could allow for more direct comparison of results between studies.

Our environmental DNA results imply the presence of fish species that have not yet been verified using traditional fishing methods, including a number of non-native species (Table 1). This is not surprising given the size of these water bodies, the sensitivity of eDNA surveys^71,72^, and the fact that aquaculture^73^ and the exotic fish trade^74^ are common in China. Two other considerations bear on our results. First, we used a single, short amplicon (133–140 bp) of the mitochondrial 16S rRNA gene where differences among closely related species may be insufficient to distinguish them. Second, definitive searches of on-line DNA data repositories like the NCBI-NT Database assume that data for all species are present and it is unlikely that there yet exists a complete catalogue of all native and non-native fish species for the Yangtze River Basin.

While we found differences between seasons in the number of species identified that may reflect life histories of local fish species and the use of different microhabitats over the annual cycle, these may also result from changes in the physicochemical properties of sampled water bodies. For example, elevated summer water temperatures can accelerate the rate of degradation of eDNA^75^. The trophic state of the water body also affects eDNA degradation; for example, eDNA decay rate of *Cyprinus carpio* was shown to be slowest in dystrophic water and fastest in oligotrophic water, and negatively correlated with dissolved organic carbon concentration^76^. Although our water samples showed little difference in temperature or dissolved oxygen between the Tian-e-Zhou Reserve or Poyang Lake (Supplementary Table S1), these abiotic factors did differ between seasons, possibly influencing not only fish diversity and behavior, but also eDNA production or persistence^23^. Further studies examining how aquatic abiotic parameters influence eDNA metabarcoding species detection and abundance estimations would be fruitful.

Another consideration for eDNA metabarcoding methods is the handling of negative controls. For example, we followed the protocol of Zou et al.^77^ and Fernández et al.^78^, only sequencing negative control samples if they showed evidence of positive PCR amplification. Our results were all negative, suggesting no contamination. However, other researchers^30,39,42^ regardless of visual confirmation of amplification, perform library preparations of negative controls for Illumina sequencing, using these data as a baseline to correct diversity metrics. Although it is often the case that negative control samples that show no PCR amplification also produce no (significant) Illumina sequencing reads^79,80^, researchers using these primers for eDNA metabarcoding fish species in the Yangtze River Basin should consider this more rigorous approach (sequencing negative control samples) when assessing amplification contamination.

## Conclusion

While our intent was to develop metabarcoding tools to non-invasively survey fish biodiversity throughout the Yangtze River Basin, our approach also shows great promise for surveying prey fish species of the YFP. Our metabarcoding approach detected many fish species that have been documented using traditional sampling practices, implying greater fish species richness than traditional netting has suggested. The Chinese government once banned fishing during the spring fish spawning season in certain areas of the Yangtze River only^81^ but now has implemented a complete 10-year fishing ban in key areas of the Yangtze River because of dramatic declines in diversity and abundance of aquatic taxa^82^. The Yangtze River is the largest river in East Asia, and the human population along its watershed exceeds 450 million people^83^. Environmental DNA metabarcoding could help fill an important but missing knowledge gap for freshwater biodiversity in China, and assist in future conservation planning across much of the country.

## Supplementary information

Supplementary file1

## Data Availability

The authors confirm all data will be deposited into FigShare upon manuscript acceptance.
