# Supplementary material for: Comparing fish prey diversity for a critically endangered aquatic mammal in a reserve and the wild using eDNA metabarcoding
Source: Sci Rep. 2020 Oct 7;10:16715. doi: 10.1038/s41598-020-73648-2 (PMC7542144; doi:10.1038/s41598-020-73648-2)
Supplement: Supplementary file 1 — Supplementary file1 [file 41598_2020_73648_MOESM1_ESM.pdf]

Supplementary information

Comparing fish prey diversity for a critically endangered aquatic mammal in a reserve and the wild using eDNA metabarcoding.

Chanjuan Qu[1,2], Kathryn A. Stewart[1,3], Rute Clemente-Carvalho[2], Jinsong Zheng[4], Yuxiang Wang[2], Cheng Gong[5], Limin Ma[1], Jianfu Zhao[1] & Stephen C. Lougheed[2]

1. State Key Laboratory for Pollution Control and Resource Reuse, College of Environmental Science and Engineering, Tongji University, Shanghai, China
2. Department of Biology, Queen's University, Kingston, Ontario, Canada
3. Institute for Biodiversity and Ecosystem Dynamics, University of Amsterdam, Amsterdam, Netherlands
4. Key Laboratory of Aquatic Biodiversity and Conservation of Chinese Academy of Sciences, Institute of Hydrobiology of Chinese Academy of Sciences, Wuhan, Hubei, China
5. Administrative Office of Hubei Yangtze Tian'eZhou Baiji National Natural Reserve, Shishou, Hubei, China

Supplementary tables

Table S1: The geographical location and water quality parameters among 15 sampling sites (Fig. 1) between two seasons (spring and summer) and sampling regions (Tian-e-Zhou Reserve and Poyang Lake).

Region	Site	Location	Sampling depth (m)		Temperature (°C)		pH		Dissolved Oxygen (mg/L)	
			spring	summer	spring	summer	spring	summer	spring	summer
Tian-e-Zhou Reserve	A	29°51′06N, 112°33′56E	12.99	12.99	13.2	23.3	7.684	7.160	10.39	2.57
	B	29°50′39N, 112°33′06E	12.99	12.99	13.0	23.4	7.634	7.161	10.35	1.63
	C	29°49′47N, 112°32′49E	7.50	7.50	15.2	27.7	8.123	6.977	13.46	7.76
	D	29°49′36N, 112°36′44E	12.99	12.99	14.8	23.9	7.604	7.103	9.83	1.04
	E	29°48′36N, 112°36′36E	3.85	3.85	16.3	28.2	7.956	6.995	10.72	7.99
Poyang Lake	a	29°14′40N, 116°07′07E	2.40	2.40	21.2	34.6	7.060	7.245	10.19	5.69
	b	29°13′45N, 116°07′15E	9.30	8.40	20.1	32.2	7.059	7.182	9.59	6.41
	c	29°13′04N, 116°10′42E	9.30	9.30	20.5	32.4	7.049	7.055	9.59	6.32
	d	29°14′01N, 116°11′08E	2.85	2.85	20.3	34.0	7.035	7.219	8.59	6.21
	e	29°14′42N, 116°11′19E	6.55	5.30	19.3	32.9	7.443	7.226	11.40	5.76
	f	29°14′41N, 116°13′05E	8.40	5.65	21.9	32.7	7.048	7.115	9.34	6.32
	g	29°12′54N, 116°13′11E	2.85	2.85	21.4	34.0	7.062	7.023	9.53	5.76
	h	29°14′21N, 116°14′20E	6.55	5.65	21.0	32.5	7.074	7.053	10.56	6.15
	i	29°12′53N, 116°16′12E	6.55	6.55	22.0	31.9	7.015	7.075	9.49	6.41
	j	29°12′30N, 116°16′53E	2.85	2.85	21.2	32.3	7.081	7.056	11.65	6.24

Table S2: A list of fish species used for designing universal primers including 28 species belonging to 4 orders, 8 families and 22 genera from the literature[1-7].

Class	Order	Family	Species	Reference	Accession Number
Actinopterygii	*Clupeiformes*	*Engraulidae*	*Coilia brachygnathus*[e]	1	KP185129
					NC_026912
			Coilia nasus	2	JX625133
					NC_019625
	Cypriniformes	*Cobitidae*	*Leptobotia taeniops*	1	KM386686
					NC_026130
					AP013304
			Parabotia fasciata	1	KM393223
					NC_026128
					AP011437
		Cyprinidae	*Acheilognathus macropterus*[e]	1	EF483935
					NC_013711
					KJ499466
			Carassius auratus[e]	3	KF147851
					EF483931
					KM659025
					KX505165
					KJ476998
					KJ874428
					KJ874429
					KJ874430
					KU146528
					NC_002079
					KJ874431
			Chanodichthys dabryi	1	KC526217
					NC_021418
					AP012111
			Chanodichthys mongolicus	1	AP009060
					NC_008683
					KF826087
					KC701385
			Ctenopharyngodon idella	4	HQ891005
					JQ231115
					EU391390

		NC_010288
		KT894100
Cyprinus carpio	3	KJ511883
		KP993137
		KU050703
		KU301745
		KF856965
		KP993136
		KU159761
		AP009047
		X61010
		NC_001606
Gobiobotia filifer	1	KP325413
		NC_029187
Hemiculter bleekeri[e]	1	KU198332
		NC_029831
Hemiculter leucisculus	5	KF956522
		AP012110
		KF647872
		NC_022929
Hypophthalmichthys molitrix	4	EU315941
		NC_010156
		JQ231114
		MF180232
		KJ671450
		KJ679503
		KJ729076
		KJ729093
		KJ729094
		KJ746939
		KJ746945
		KJ746946
		KJ746947
		KJ746948
		KJ746952
		KJ746953
		KJ746954
		KJ746956
		KJ671449

		KJ729092
		KJ746938
		KJ746940
		KJ746943
		KJ746944
		KJ746949
		KJ746950
		KJ746951
		KJ746955
		KJ746957
		KJ746960
		KJ746961
		MF180230
		KP013119
Hypophthalmichthys nobilis	4	EU343733
		NC_010194
		KJ679504
		KJ679505
		KJ710363
		KJ729077
		KJ729080
		KJ729081
		KJ729082
		KJ729084
		KJ729088
		KJ729089
		KJ729091
		KJ729096
		KJ746935
		KJ746936
		KJ746937
		KJ746942
		KJ746959
		KJ746962
		KJ746963
		KJ756343
		HM162839
		KJ710362
		KJ729078

			KJ729079
			KJ729083
			KJ729085
			KJ729086
			KJ729087
			KJ729090
			KJ729095
			KJ729097
			KJ746958
			KJ746966
			KJ746941
			AP011217
	Mylopharyngodon piceus	5	MF687109
			MF687110
			MF687111
			MF687112
			MF687113
			MF687114
			MF687115
			MF687116
			MF687117
			MF687118
			MF687119
			MF687120
			MF687121
			MF687122
			MF687123
			MF687124
			MF687125
			MF687126
			MF687127
			MF687128
			MF687129
			MF687130
			MF687131
			MF687132
			MF687133
			MF687134
			MF687135

				MF687136
				MF687137
				DQ026435
				AP011216
				EU979306
				EU979305
				EU979307
				NC_011141
		Parabramis pekinensis[e]	4	KF857485
				JX242531
				NC_022678
		Pseudobrama simoni	1	KF537571
				NC_022852
				AP011364
		Pseudolaubuca engraulis	1	KC429670
				NC_020462
		Squalidus argentatus[e]	1	KF819452
				KF801059
				NC_023336
				KF926824
		Xenocypris davidi	6	GQ289558
				NC_013072
				KF039718
Perciformes	*Channidae*	*Channa argus*	7	GU937112
				NC_015191
				JX978723
				KC823605
				AP006041
	Cichlidae	*Oreochromis mossambicus*	2	AY597335
				NC_007231
	Gobiidae	*Rhinogobius giurinus*[e]	1	KF371534
				NC_022692
				KP892753
				KU871066
Siluriformes	*Bagridae*	*Tachysurus argentivittatus*	1	KX164404
				NC_030538
		Tachysurus fulvidraco[e]	6	HM641815
				NC_015888

			KC287172
	Tachysurus nitidus	1	KC822643
			HM746659
			NC_014859
Siluridae	*Silurus asotus*[e]	7	JN116720
			JX087351
			JX256247
			AP012022
			NC_015806

Note: (1) [e]9 fish species that we have their tissue samples. (2) The Latin name of fish species and the higher level of classification are based on https://www.fishbase.se/.

Table S3: Target sequences of 19 species of fishes for making synthetic DNA solutions.

Species	Sequences
Channa argus	ATTAACCCTCGTACCTTTTGCATCATGATTTAGCCAGTAAGCCCAAGCAAAGAGCACTTTAGTTTGACCCCCCGAAACTAAGTGA<br>GCTACTCCAAGACAGCCTATTTTAGGGCCAACCCGTCTCTGTGGCAAAAGAGTGGGAAGAGCTTTGAGTA
Chanodichthys dabryi,<br>*Chanodichthys mongolicus,*<br>*Pseudobrama simoni*	ACTAAACCTTGTACCTTTTGCATCATGATTTAGCCAGCACCCTCAAGCAAAGAGACCTTTAGTTTGAAACCCCGAAACCAGGTGA<br>GCTACCCCGAGACAGCCTATTTAAATTTAGGGCTAACCCGTCTCTGTGGCAAAAGAGTGGGAAGAGCTCCGGGTA
Coilia nasus	ACACCCCTCGTACCTTTTGCATCATGATTCAGCTAGTAAAATACATGCAAAGAGACCTCTAGTTTGTAACCCCGAAACCCGACGA<br>GCTACTCCGGGACAGCCTATCAAGGGCCAACCCGTCTCTGTGGCAAAAGAGTGGGAAGATCCCCGAGTA
Ctenopharyngodon idella	TAAACCTTGTACCTTTTGCATCATGATTTAGCCAGCACCCTCAAGCAAAGAGACCTTTAGTTTGAAACCCCGAAACCAGGTGAGC<br>TACCCCGAGACAGCCTATTTAAATTTAGGGCTAACCCGTCTCTGTGGCAAAAGAGTGGGAAGAGCTCCGGGTA
Cyprinus carpio	ATTAAACCTCGTACCTTTTGCATCATGATTTAGCCAGTACACCCAAGCAAAGAGACCTTTAGTTTGAAACCCCGAAACCAGGTGA<br>GCTACCCCGAGACAGCCTATTAAGGGCCAACCCGTCTCTGTGGCAAAAGAGTGGGAAGAGCTCCGGGTA
Gobiobotia filifer	ATTAAACCTTGTACCTTTTGCATCATGATTTAGCCAGTACCTCCAAGCAAAGAGACCTTTAGTTTGAAACCCCGAAACCAGGTGA<br>GCTACCCCGAGACAGCCTATATAACTTAGGGCTAACCCGTCTCTGTGGCAAAAGAGTGGAAGAGCTCCGGGGT
Hemiculter leucisculus	ACTAAACCTTGTACCTTTTGCATCATGATTTAGCCAGCACCCTCAAGCAGAGAGACCTTTAGTTTGAAACCCCGAAACCAGGTGA<br>GCTACCCCGAGACAGCCTATTTAAATTTAGGGCTAACCCGTCTCTGTGGCAAAAGAGTGGGAAGAGCTCCGGGTA
Hypophthalmichthys molitrix,<br>*Hypophthalmichthys nobilis*	ACTAAACCTTGTACCTTTTGCATCATGATTTAGCCAGCACCCTCAAGCAAAGAGACCTTTAGTTTGAAACCCCGAAACCAGGTGA<br>GCTACCCCGAGACAGCCTATTAAAATTTAGGGCTAACCCGTCTCTGTGGCAAAAGAGTGGGAAGAGCTCCGGGTA
Leptobotia taeniops	ACTAAAACTCGTACCTTTTGCATCATGATTTAGCCAGCACCCTCAAGCAAAGAGACCTTTAGTTTGAAACCCCGAAACCAAGTGA<br>GCTACCCCGAGACAGCCTATATGGGCCAACCCGTCTCTGTGGCAAAAGAGTGGGAAGAGCTCTGGGTA
Mylopharyngodon piceus	ATTAAACCTTGTACCTTTTGCATCATGATTTAGCAAGCACCCTCAAGCAAAGAGACCTTTAGTTTGAAACCCCGAAACCAGGTGA<br>GCTACCCCGAGACAGCCTATTTAAATTTAGGGCTAACCCGTCTCTGTGGCAAAAGAGTGGGAAGAGCTCTGGGTA
Oreochromis mossambicus	ACCGCCCCTCGTACCTTTTGCATCATGATTTAGCCAGAAATCTCTTAAGCAAAAAGCATTATAGTTTAATACCCCGAAACTAAGC<br>GAGCTACTCCAAGACAGTCTAATTTATAGGACCGCCCCGTCTCTGTGGCAAAAGAGTGGGAAGAACTTTGAGTA
Parabotia fasciata	ATTAAAACTCGTACCTTTTGCATCATGATTTAGCCAGCACCCTCAAGCAAAGAGACCTTTAGTTTGAAACCCCGAAACCAAGTGA<br>GCTACCCCGAGACAGCCTTCATGGGCCAACCCGTCTCTGTGGCAAAAGAGTGGGAAGAGCTCTGGGTA

Pseudolaubuca engraulis	ACTAAACCTTGTACCTTTTGCATCATGATTTAGCCAGCACCCTCAAGCAAAGAGACCTTTAGTTTGAAACCCCGAAACCAGGTGA<br>GCTACCCCGAGACAGCCTATTTAAATTTAGGGCTAACCCGTCTCTGTGGCAAAAGAGTGGGGAGAGCTCCGGGTA
Tachysurus argentivittatus	ATTAAACCTCGTACCTTTTGCATCATGATTTAGCTAGACCTTCTGAGCAAAGAGTACTTTAGTTCAAAACCCCGAAACTACGTGA<br>GCTACCCCGAAACAGCCTATCAATTAGGGCCAGCCCGTCTCTGTGGCAAAAGAGTGGGAAGATTACCGGGTA
Tachysurus nitidus	ATTAAACCTCGTACCTTTTGCATCATGATTTAGCTAGACCTTCTGAGCAAAGAGTACTTTAGTTCAAAACCCCGAAACTACGTGA<br>GCTACCCCGAAACAGCCTATAAATTAGGGCCAACCCGTCTCTGTGGCAAAAGAGTGGGAAGATTTCCGGGTA
Xenocypris davidi	ACTAAACCTTGTACCTTTTGCATCATGATTTAGCCAGCACCCTCAAGCAAGGAGACCTTTAGTTTGAAACCCCGAAACCAGGTGA<br>GCTACCCCGAGACAGCCTATTTAAATTAGGGCTAACCCGTCTCTGTGGCAAAAGAGTGGGAAGAGCTCCGGGTA

Table S4: Primer sequences for library preparation.

The first-round PCR primers

Forward:	ACACTCTTTCCCTACACGA + CGCTCTTCCGATCT + 16S_eDNAF sequences
Reverse:	GTGACTGGAGTTCAGACGTG + TGCTCTTCCGATCT + 16S_eDNAR sequences

The second-round PCR primers

Forward:	AATGATACGGCGACCACCGAGATCTACAC + i5 bases + ACACTCTTTCCCTACACGA
Reverse:	CAAGCAGAAGACGGCATACGAGAT + i7 bases + GTGACTGGAGTTCAGACGTG

Sample number	i7 index name	i7 bases	i5 index name	i5 bases
A41	D701	CGAGTAAT	D501	TATAGCCT
A42	D702	TCTCCGGA	D501	TATAGCCT
A43	D703	AATGAGCG	D501	TATAGCCT
B41	D704	GGAATCTC	D501	TATAGCCT
B42	D705	TTCTGAAT	D501	TATAGCCT
B43	D706	ACGAATTC	D501	TATAGCCT
C41	D707	AGCTTCAG	D501	TATAGCCT
C42	D708	GCGCATTA	D501	TATAGCCT
C43	D709	CATAGCCG	D501	TATAGCCT
D41	D710	TTCGCGGA	D501	TATAGCCT
D42	D711	GCGCGAGA	D501	TATAGCCT
D43	D712	CTATCGCT	D501	TATAGCCT
E41	D701	CGAGTAAT	D502	ATAGAGGC
E42	D702	TCTCCGGA	D502	ATAGAGGC
E43	D703	AATGAGCG	D502	ATAGAGGC
d41	D704	GGAATCTC	D502	ATAGAGGC
d42	D705	TTCTGAAT	D502	ATAGAGGC
d43	D706	ACGAATTC	D502	ATAGAGGC
e41	D707	AGCTTCAG	D502	ATAGAGGC
e42	D708	GCGCATTA	D502	ATAGAGGC
e43	D709	CATAGCCG	D502	ATAGAGGC
c41	D710	TTCGCGGA	D502	ATAGAGGC
c42	D711	GCGCGAGA	D502	ATAGAGGC
c43	D712	CTATCGCT	D502	ATAGAGGC
b41	D701	CGAGTAAT	D503	CCTATCCT
b42	D702	TCTCCGGA	D503	CCTATCCT
b43	D703	AATGAGCG	D503	CCTATCCT
a41	D704	GGAATCTC	D503	CCTATCCT
a42	D705	TTCTGAAT	D503	CCTATCCT
a43	D706	ACGAATTC	D503	CCTATCCT
h41	D707	AGCTTCAG	D503	CCTATCCT

h42	D708	GCGCATTA	D503	CCTATCCT
h43	D709	CATAGCCG	D503	CCTATCCT
g41	D710	TTCGCGGA	D503	CCTATCCT
g42	D711	GCGCGAGA	D503	CCTATCCT
g43	D712	CTATCGCT	D503	CCTATCCT
j41	D701	CGAGTAAT	D504	GGCTCTGA
j42	D702	TCTCCGGA	D504	GGCTCTGA
j43	D703	AATGAGCG	D504	GGCTCTGA
i41	D704	GGAATCTC	D504	GGCTCTGA
i42	D705	TTCTGAAT	D504	GGCTCTGA
i43	D706	ACGAATTC	D504	GGCTCTGA
f41	D707	AGCTTCAG	D504	GGCTCTGA
f42	D708	GCGCATTA	D504	GGCTCTGA
f43	D709	CATAGCCG	D504	GGCTCTGA
A71	D710	TTCGCGGA	D504	GGCTCTGA
A72	D711	GCGCGAGA	D504	GGCTCTGA
A73	D712	CTATCGCT	D504	GGCTCTGA
B71	D701	CGAGTAAT	D505	AGGCGAAG
B72	D702	TCTCCGGA	D505	AGGCGAAG
B73	D703	AATGAGCG	D505	AGGCGAAG
C71	D704	GGAATCTC	D505	AGGCGAAG
C72	D705	TTCTGAAT	D505	AGGCGAAG
C73	D706	ACGAATTC	D505	AGGCGAAG
D71	D707	AGCTTCAG	D505	AGGCGAAG
D72	D708	GCGCATTA	D505	AGGCGAAG
D73	D709	CATAGCCG	D505	AGGCGAAG
E71	D710	TTCGCGGA	D505	AGGCGAAG
E72	D711	GCGCGAGA	D505	AGGCGAAG
E73	D712	CTATCGCT	D505	AGGCGAAG
d71	D701	CGAGTAAT	D506	TAATCTTA
d72	D702	TCTCCGGA	D506	TAATCTTA
d73	D703	AATGAGCG	D506	TAATCTTA
e71	D704	GGAATCTC	D506	TAATCTTA
e72	D705	TTCTGAAT	D506	TAATCTTA
e73	D706	ACGAATTC	D506	TAATCTTA
c71	D707	AGCTTCAG	D506	TAATCTTA
c72	D708	GCGCATTA	D506	TAATCTTA
c73	D709	CATAGCCG	D506	TAATCTTA
b71	D710	TTCGCGGA	D506	TAATCTTA
b72	D711	GCGCGAGA	D506	TAATCTTA
b73	D712	CTATCGCT	D506	TAATCTTA
a71	D701	CGAGTAAT	D507	CAGGACGT
a72	D702	TCTCCGGA	D507	CAGGACGT

a73	D703	AATGAGCG	D507	CAGGACGT
h71	D704	GGAATCTC	D507	CAGGACGT
h72	D705	TTCTGAAT	D507	CAGGACGT
h73	D706	ACGAATTC	D507	CAGGACGT
g71	D707	AGCTTCAG	D507	CAGGACGT
g72	D708	GCGCATTA	D507	CAGGACGT
g73	D709	CATAGCCG	D507	CAGGACGT
j71	D710	TTCGCGGA	D507	CAGGACGT
j72	D711	GCGCGAGA	D507	CAGGACGT
j73	D712	CTATCGCT	D507	CAGGACGT
i71	D701	CGAGTAAT	D508	GTACTGAC
i72	D702	TCTCCGGA	D508	GTACTGAC
i73	D703	AATGAGCG	D508	GTACTGAC
f71	D704	GGAATCTC	D508	GTACTGAC
f72	D705	TTCTGAAT	D508	GTACTGAC
f73	D706	ACGAATTC	D508	GTACTGAC

Note: (1) Site A, B, C, D and E are from Tian-e-Zhou Baiji National Nature Reserve; site a, b, c, d, e, f, g, h, i and j are from Poyang Lake. (2) Sample number A41, A42 and A43 represent 3 PCR replicates of the eDNA sample collected at sampling site A in spring, respectively; sample number A71, A72 and A73 represent 3 PCR replicates of the eDNA sample collected at sampling site A in summer, respectively; other sample numbers have similar meanings.

Table S5: Taxonomic composition and read numbers of the 101 species of fishes from 90 libraries identified in HiSeq analyses of eDNA samples

Class	Order	Family	Species	A41	A42	A43	B41	B42	B43	C41	C42	C43	D41	D42	D43	E41	E42	E43	a41	a42	a43	b41	b42	b43	c41	c42	c43	d41	d42	d43	e41	e42	e43	f41	f42	f43	g41	g42	g43	h41	h42	h43	i41	i42	i43	j41	j42	j43
Actinopterygii	Cypriniformes	Cyprinidae	*Acanthorhodeus chankaensis*	72	89	79	66	76	58	76	66,327	65	174	32,569	48	23	46	17	3,306	1,123	4,209	15	23	14	23,955	52		43	29	20	34	129	18	8,952	14,090	16,920	2,914	84	14	7,891	4,312	27	13,495	7,397	1,588	45	4,713	44
Actinopterygii	Cypriniformes	Cyprinidae	*Acheilognathus imberbis*		10	10	11										13	14			3,048		13	16						11				32	54	35			10				9,073	12,643	7,956	16,041	8,882	15,539
Actinopterygii	Cypriniformes	Cyprinidae	*Acheilognathus meridianus*																															12	10													
Actinopterygii	Cypriniformes	Cyprinidae	*Acrossocheilus fasciatus*		25		117		263					284		301	434	588	215	176	131	2,656	224	666	561	82	168	120	61	183	109	2,042	298	178	128	154	323	579	252	688	429	255	154	159	212	186	256	146
Actinopterygii	Cypriniformes	Cyprinidae	*Acrossocheilus kreyenbergii*	1,583	61	59	72	88	151	22	102	33	75	192	48	842	15,495	5,666	8,242	11,056	13,018	91,267	41,678	62,613	25,806	30,193	32,462	253	13,526	335	201	119,801	7,679	13,058	14,116	9,960	26,643	51,683	28,216	2,028	11,511	6,068	5,273	9,336	4,869	2,369	4,996	5,133
Actinopterygii	Cypriniformes	Cyprinidae	*Acrossocheilus parallens*		42	38	163		341					443		520	712	11,581	243	247	165	1,567	309	791	810	102	711	189	105	265	176	2,499	639	254	173	201	375	766	6,036	1,165	540	341	230	257	265	271	366	175
Actinopterygii	Cypriniformes	Cyprinidae	*Ancherythroculter kurematsui*	28			81		117	118		24	134	83	19	93	43	66	101	81	103	273	100	44		31	34		16	250		62		28	24	10		91	41		64			13	10		11	
Actinopterygii	Cypriniformes	Cyprinidae	*Ancherythroculter lini*				13		24	50		10	53	21		19	13	28	19	25	30	57	30	10						15		20			11			17			14							
Actinopterygii	Cypriniformes	Cyprinidae	*Balantiocheilos melanopterus*																		17	35		373			24					19			21	12		23			10	18	11	10				
Actinopterygii	Cypriniformes	Cyprinidae	*Barbodes banksi*		37	23	104		266		10			240		262	483	634	172	129	98	916	178	432	413	64	125	89	68	167	77	1,279	264	150	102	121	213	440	257	500	349	181	129	133	154	168	210	131
Actinopterygii	Cypriniformes	Cyprinidae	*Barbodes binotatus*						18					16			34	87		10		61		45	24							110					10	24	15	36	12							
Actinopterygii	Cypriniformes	Cyprinidae	*Barbus ciscaucasicus*				40		83					77		100	151	261	71	59	35	548	74	173	156	15	51	41	19	52	37	596	157	76	47	56	84	144	64	235	141	76	59	43	72	70	75	53
Actinopterygii	Cypriniformes	Cyprinidae	*Carassius auratus*														10	186	13	13	12	85	27	65	17	31	38		15			144		23	18	18	28	50	23		16			14	960			
Actinopterygii	Cypriniformes	Cyprinidae	*Carassius carassius*																	13	10	57	68	45	25	20	19					77		15		11	18	38	19									
Actinopterygii	Cypriniformes	Cyprinidae	*Chanodichthys erythropterus*	28		18	53	13	118	169		48	219	84	33	93	51	77	84	2,471	92	266	102	46		31	26			76		78		28	20			43	38		65			15				
Actinopterygii	Cypriniformes	Cyprinidae	*Clinostomus elongatus*[f]																																			14										
Actinopterygii	Cypriniformes	Cobitidae	*Cobitis elongatoides*																	16	22																											
Actinopterygii	Cypriniformes	Cobitidae	*Cobitis lutheri*																																													
Actinopterygii	Clupeiformes	Engraulidae	*Coilia lindmani*							75						28			130	127	1,026	149	80	199	171	114	106	101	156	113	237	877	248	560	331	216	66	252	68	2,471	184	151	1,997	2,024	524	388	528	438
Actinopterygii	Clupeiformes	Engraulidae	*Coilia mystus*							12										13	17	26	22	35	43	12	17	10	21	19	50	187	54	28	58	39		15		39	29	13	76	78	95	79	93	62
Actinopterygii	Siluriformes	Bagridae	*Coreobagrus brevicorpus*	18					28										41	75	68	666	135	223	413	163	162	73	66	144	132	65	128	17			314	825	269	120	136	139		28	32		12	
Actinopterygii	Cypriniformes	Cyprinidae	*Cyclocheilichthys janthochir*													13																																
Actinopterygii	Cypriniformes	Cyprinidae	*Cyprinus acutidorsalis*[f]																			26										33						10		21								
Actinopterygii	Cypriniformes	Cyprinidae	*Cyprinus carpio 'Furong' x Carassius auratus red var.*[f]															12														10																
Actinopterygii	Cypriniformes	Cyprinidae	*Cyprinus carpio 'mirror' x Cyprinus carpio 'singuonensis'*						33					15		11	30	43				87	12	41	34							126	24				23	32	27	43	39	10					32	
Actinopterygii	Cypriniformes	Cyprinidae	*Cyprinus megalophthalmus*						27					36		56	78	85	11			163	32	76	66			11		24		201	40		17	34	31	68	31	76	50	27	25	15	13	26	43	29
Actinopterygii	Cypriniformes	Cyprinidae	*Dawkinsia tambraparniei*															12				10		11								29																
Actinopterygii	Cypriniformes	Cyprinidae	*Diptychus maculatus*[f]															13																														
Actinopterygii	Cypriniformes	Cyprinidae	*Discherodontus ashmeadi*						16					18		23	24	30	10			89	26	28	39					19		101	20	11		13	23	35	26	40	33		14			14	23	
Actinopterygii	Cypriniformes	Cyprinidae	*Elopichthys bambusa*	27	29	22	27	21	18	46,320	816	22	23	45	23	6,093									13	29	12	11			38	16		22				24		90								
Actinopterygii	Cypriniformes	Cyprinidae	*Eremichthys acros*																												13	89																
Actinopterygii	Esociformes	Esocidae	*Esox lucius*[f]																														9,225															
Actinopterygii	Cypriniformes	Cyprinidae	*Gnathopogon strigatus*	17													60						31					50	49	350	53	635	174							76	74	99		21	24	29		30
Actinopterygii	Cypriniformes	Cyprinidae	*Gobiobotia filifer*[f]																																						11							
Actinopterygii	Cypriniformes	Cyprinidae	*Gobiobotia macrocephala*	60	51	42		26		103	339	98				437	496	438	11	2,544	10	49	109	53	392	450	289	412	391	961	100,655	380,934	106,203	39,960	19,349	21,840	11			245	367	9,414	22,597	38,577	9,898	6,010	2,803	3,901
Actinopterygii	Cypriniformes	Cyprinidae	*Hemibarbus labeo*													25	24	139																														
Actinopterygii	Cypriniformes	Cyprinidae	*Hemibarbus umbrifer*												52	22	28	68																														
Actinopterygii	Cypriniformes	Cyprinidae	*Hemiculter leucisculus*	23	123	75	299	420	389	181	150	46	174	216	19	160	43	69	97	89	92	258	168	40		19	25		30	84		65		40	33	10	24	108	84		57			22	34	13	39	
Actinopterygii	Cypriniformes	Cyprinidae	*Hemiculterella sauvagei*										45	25				11																														
Actinopterygii	Cypriniformes	Cyprinidae	*Hemitremia flammea*[f]																																10			10										
Actinopterygii	Cypriniformes	Cyprinidae	*Hypophthalmichthys molitrix*	21	10	12	226	272	123		73	89	446	949	214	156	268	239	39	25	43			18	51	14					29	73						20						12				
Actinopterygii	Beloniformes	Hemiramphidae	*Hyporhamphus intermedius*													11			15		24																											
Actinopterygii	Cypriniformes	Cyprinidae	*Labeo cyclorhynchus*[f]																																													
Actinopterygii	Cypriniformes	Cyprinidae	*Labeo pierrei*[f]																								11																					
Actinopterygii	Cypriniformes	Cyprinidae	*Leuciscus oxyrrhis*[f]																																													
Actinopterygii	Cypriniformes	Cyprinidae	*Margariscus margarita*							12	47									56											496	2,697	423	246	211	177						34	286	451	102	46	34	38
Actinopterygii	Perciformes	Odontobutidae	*Micropercops swinhonis*[f]															6,396																														
Actinopterygii	Perciformes	Centrarchidae	*Micropterus salmoides*								17					12	12	19							18	25			23	11	11	31,466	16		14						15			2,436				
Actinopterygii	Perciformes	Gobiidae	*Mugilogobius myxodermus*[f]																																													
Actinopterygii	Salmoniformes	Salmonidae	*Oncorhynchus formosanus*	596	889	471	423	390	208	1,025	1,304	541	726	520	336	66	69	172	45	70	48	91	38	35	458	192	212	78	82	373	24	156	31	108	109	95	22	73	34	30	68	38		12	10	11	17	973
Actinopterygii	Cypriniformes	Cyprinidae	*Onychostoma barbatulum*				57		97					123		135	225	304	75	74	38	399	94	234	203	22	57	49	31	85	53	902	101	95	55	42	105	214	97	314	160	71	73	84	88	72	111	57
Actinopterygii	Cypriniformes	Cyprinidae	*Onychostoma gerlachi*[f]																					10									12															
Actinopterygii	Cypriniformes	Cyprinidae	*Osteobrama cotio*				30		80					90		110	140	196	77	148	52	503	104	238	177	31	55	43	37	67	28	658	141	94	226	67	126	197	131	335	202	97	116	106	139	153	156	94
Actinopterygii	Siluriformes	Bagridae	*Pelteobagrus eupogon*	34					61			78							125	183	150	1,780	270	527	954	416	338	191	128	280	318	119	556	50			791	1,969	583	298	378	325	44	82	69		39	
Actinopterygii	Siluriformes	Bagridae	*Pelteobagrus intermedius*	29			269		35										68	6,275	86	1,448	370	601	703	270	457	367	103	288	253	53	229	30			463	1,376	375	242	224	225	27	52	48		38	
Actinopterygii	Perciformes	Percidae	*Percina maculata*[f]																408																													
Actinopterygii	Perciformes	Percidae	*Percina rex*[f]																313																													
Actinopterygii	Cypriniformes	Cyprinidae	*Percocypris regani*[f]																														12															
Actinopterygii	Cypriniformes	Cyprinidae	*Platygobio gracilis*																												11	36																
Actinopterygii	Cypriniformes	Cyprinidae	*Procypris rabaudi*																																12	10												
Actinopterygii	Salmoniformes	Salmonidae	*Prosopium cylindraceum*	2,108	3,738	1,598	1,250	1,002	405	4,845	2,376	1,440	1,335	1,278	606	113	104	310	113	203	249	272	990	97	1,854	631	999	760	338	900	88	480	166	296	301	268	124	158	64	89	215	121	15	29	48	19	63	35
Actinopterygii	Cypriniformes	Cyprinidae	*Pseudaspius leptocephalus*																																17									14				
Actinopterygii	Siluriformes	Bagridae	*Pseudobagrus brevicaudatus*[f]																					158																								
Actinopterygii	Siluriformes	Bagridae	*Pseudobagrus ondon*	29	17				85				23			34	65	31	117	160	98	1,319	15,988	432	25,792	350	381	158	152	238	266	111	260	33			653	1,627	482	332	308	262	30	57	54		45	
Actinopterygii	Siluriformes	Bagridae	*Pseudobagrus trilineatus*[f]																	15																												
Actinopterygii	Siluriformes	Bagridae	*Pseudobagrus vachellii*				93		45							25	26	24	4,910	2,742	1,745	5,303	757	1,622	713	416	207	506	202	1,147	929	398	1,162	180	7,258	45	1,704	5,264	1,211	1,139	1,718	660	87	124	146	40	66	10
Actinopterygii	Cypriniformes	Cyprinidae	*Pseudocrossocheilus tridentis*[f]															11									1,258					17																
Actinopterygii	Cypriniformes	Cyprinidae	*Pseudohemiculter dispar*	11			25		42	41		12	71	22		23	14	28	24	18	22	98	24	19			11			32		30						13	10		13							
Actinopterygii	Cypriniformes	Cyprinidae	*Pseudolaubuca engraulis*	5,399	327	2,206	15,396	2,218	30,985	39,574	527	10,417	43,421	21,019	6,065	23,145	12,403	22,425	20,748	17,291	20,717	69,052	28,203	7,968	281	5,996	6,609	173	155	20,176	185	18,483	218	4,374	5,331	696	393	11,670	6,312	1,771	15,443	182	232	4,643	889	215	192	911
Actinopterygii	Cypriniformes	Cyprinidae	*Pseudolaubuca sinensis*[f]																	20																		65										
Actinopterygii	Cypriniformes	Cyprinidae	*Ptychocheilus umpquae*																												76	676																
Actinopterygii	Cypriniformes	Cyprinidae	*Puntioplites waandersi*				10		10							27	41	37	24	11	11	118		60	32		319			12		117	24	11				37		52	24	14	11	11	12	23	32	
Actinopterygii	Cypriniformes	Cyprinidae	*Rhinichthys osculus*[f]																													15																
Actinopterygii	Perciformes	Gobiidae	*Rhinogobius brunneus*					10																					11							712												
Actinopterygii	Perciformes	Gobiidae	*Rhinogobius cliffordpopei*																	12													28															
Actinopterygii	Perciformes	Gobiidae	*Rhinogobius giurinus*		95	22	133	46	108	77	119	44	2,417	157	137	32	582	61	26,129	20,264	39,030	66	142	64	257	36,485	146	168	92	137	95	168	70	124	45,289	10,853	197	211	146	103	149	78	174	11,960	132	3,175	152	82
Actinopterygii	Cypriniformes	Cyprinidae	*Rutilus rutilus*																												33	223																
Actinopterygii	Salmoniformes	Salmonidae	*Salmo salar*		42	20	614	750	464	4,914	11	355	84	402					264	586	686		1,544		3,287	88	147	644	69		85		637	21			358							21	118			
Actinopterygii	Salmoniformes	Salmonidae	*Salvelinus confluentus*		37					44	17			14																																		
Actinopterygii	Cypriniformes	Cyprinidae	*Sarcocheilichthys kiangsiensis*[f]																																										14			
Actinopterygii	Cypriniformes	Cyprinidae	*Sarcocheilichthys lacustris*																													87			64	15												
Actinopterygii	Cypriniformes	Cyprinidae	*Sarcocheilichthys nigripinnis*[f]														3,996													10															4,744			
Actinopterygii	Cypriniformes	Cyprinidae	*Sarcocheilichthys sinensis*																															918	5,852	2,040												
Actinopterygii	Cypriniformes	Cyprinidae	*Saurogobio immaculatus*			29												21	28		101			716			49				23	160			79	30				87	22	48	115	82				18
Actinopterygii	Cypriniformes	Cyprinidae	*Schizothorax esocinus*[f]																			34																										
Actinopterygii	Cypriniformes	Cyprinidae	*Schizothorax plagiostomus*				28		56					58		36	123	153	58	38	26	139	43	180	112	12	51	23		37	13	552	30	47	13		59	136	61	166	80	31	17	30	45	11	36	38
Actinopterygii	Cypriniformes	Cyprinidae	*Semotilus atromaculatus*	30	12	29			20	31	123	15	10		154	150	496	343		895	17		77		52	61	53	90	74	822	6,557	42,409	376	71	126	104				130	190	117	111	201	149	112	74	118
Actinopterygii	Siluriformes	Siluridae	*Silurus asotus*							11									33	56	48	11		11				24	30	33				27	6,322	1,878	123	256	2,345	14		1,552			26	11		10
Actinopterygii	Siluriformes	Siluridae	*Silurus lanzhouensis*																																			93										
Actinopterygii	Cypriniformes	Cyprinidae	*Sinibrama macrops*	14			65	23	86	66		16	148	69	12	90	54	82	66	35	41	167	73	14		11	14		11	48		64			10			55			44						13	
Actinopterygii	Cypriniformes	Cyprinidae	*Sinibrama taeniatus*[f]																																													
Actinopterygii	Cypriniformes	Cyprinidae	*Spinibarbus denticulatus*				26		74					71		87	186	222	60	41	26	271	49	116	100	11	21	10	11	44	24	452	94	44	25	40	54	137	55	149	96	44	54	103	47	37	52	27
Actinopterygii	Cypriniformes	Cyprinidae	*Squalidus argentatus*	101	96	115	125,604	143	128	146	146	91	103	116	99	15	137	12,843	3,600	65	10			14	16	14	24	153	81	12	46	32	21	38,531	22,079	12,211	50	22,479	13,697	68	60	46	123	6,069	51	59	3,655	61
Actinopterygii	Cypriniformes	Cyprinidae	*Squalidus multimaculatus*	18											50	44	136	209		10			29					28	28	341	95	972	107	21	13	21				76	43	65	18	20		25	13	18
Actinopterygii	Cypriniformes	Cyprinidae	*Squalidus wolterstorffi*														48	19																														
Actinopterygii	Cypriniformes	Cyprinidae	*Squalius lepidus*																													10						22	10						20			
Actinopterygii	Siluriformes	Bagridae	*Tachysurus argentivittatus*[f]																					109																								
Actinopterygii	Siluriformes	Bagridae	*Tachysurus nitidus*	288	187	239	86	93	12,486	242	773	177	78	272	56	1,351	1,192	1,210	32,735	39,289	22,602	240,749	109,120	144,384	27,521	15,636	815	64,196	32,517	57,579	147,149	801,326	176,255	78,434	61,997	58,456	75,338	219,570	57,323	98,129	122,390	31,012	38,469	42,173	47,819	31,057	43,720	52,906
Actinopterygii	Cypriniformes	Cyprinidae	*Tinca tinca*				841										323	362	42												13	132		77	66			67	45					47				
Actinopterygii	Cypriniformes	Cyprinidae	*Toxabramis houdemeri*										60	40				12																				98										
Actinopterygii	Cypriniformes	Cyprinidae	*Xenocypris davidi*										128				199	109																														
**Total read numbers**				10,534	5,917	5,107	146,222	5,591	47,548	98,154	73,277	13,621	49,947	59,564	7,971	34,783	39,107	65,963	102,882	106,849	108,073	421,146	201,356	223,666	115,519	92,089	46,503	69,064	48,727	85,760	258,712	1,413,298	306,140	187,276	204,081	137,410	111,660	321,182	118,471	119,288	160,285	51,841	93,135	139,604	81,476	60,775	71,590	81,077

Note: (1) Site A, B, C, D and E are from Tian-e-Zhou Baiji National Nature Reserve; site a, b, c, d, e, f, g, h, i and j are from Poyang Lake. (2) Sample number A41, A42 and A43 represent 3 PCR replicates of the eDNA sample collected at sampling site A in spring, respectively; sample number A71, A72 and A73 represent 3 PCR replicates of the eDNA sample collected at sampling site A in summer, respectively; other sample numbers have similar meanings. (3) The Latin name of fish species and the higher level of classification are based on https://www.fishbase.se/. (4) [f]26 fish species removed.

A71	A72	A73	B71	B72	B73	C71	C72	C73	D71	D72	D73	E71	E72	E73	a71	a72	a73	b71	b72	b73	c71	c72	c73	d71	d72	d73	e71	e72	e73	f71	f72	f73	g71	g72	g73	h71	h72	h73	i71	i72	i73	j71	j72	j73
91	70	34				22	3,187	11		69		35	34		19	21	18	2,550	2,966	35	22	104	24	3,733	4,007	2,964	22	5,630	20	9,030	22	3,603	18	68	17	22	6,044	13	29	15	16	7,773	31	
66	31	45																10	2,437	10	2,298	11	10	3,067	12	23	12		12																
	53	14			48	31	44	76	42				12						527	325	288	278	439	336	158	149	119	77	133	155	163	282	211				303	126	432	184	261	233	405	363	252
101	131	2,447	47	30	54	1,010	3,231	2,019	27	80	23	987	500	51	139	16,614	2,402	49,772	37,181	39,551	8,914	4,178	18,454	8,299	10,084	6,649	54,943	59,800	71,577	43,943	17,855	30,013	5,438	249	16,722	19,861	49,503	18,018	13,068	11,958	173	15,397	15,047	3,994
10	72	46			56	48	67	251	84					11				251	356	389	7,234	702	400	208	203	229	94	127	159	232	317	202				434	143	314	304	347	227	825	491	352
		14	40		15	10						31	31	23	153		70	30	49	60			30	13			83	32	11	83	86	138	268	34	194	43	68	69	47	60	47	61	25	27
			14									83		14	43		20	19	15	12							11	10		12	39	29	31	15	56	13		11			17	27		
																		10		10				32	25	19	27	24	37	16	12	21				20	22		18			26		
	66	34			56	40	46	94	58	10		12						113	344	199	178	216	221	120	337	107	61	145	154	119	196	129				287	108	172	156	170	262	380	265	219
							10													15	23	11	22																		27	16	
	21						10	32										57	91	59	95	106	102	32	90	61	10	23	23	41	71	47				145	32	65	77	81	53	143	97	90
							11									22	11	39	24	29			12			13	55	59	75	35	13	24			14	77	41		11	11		10	23	
																18		42	54	21	16		20		24		31	43	66	36	20	23			18	24	31		15			16		
		20	46		19	16	13					40	26	38	171		63	47	48	43			29				70	46	21	76	117	56	120	68	203	237	53	34	42	29	56	110	25	14
															14	14																				14							12	
182	216	206	102	147	158	209	259	145	70	19	43	86	82	91	43		39	350	270	286	623	624	516	432	700	384	88	41	73	77	113	155	71	42	171	73	84	33	379	334	349	89	372	198
46	48	54	30	38	24	50	55	41	11		14	13	30	11			10	162	86	35	119	100	84	52	79	53	10		13	18	17	24	25	23	36	17	24		96	79	78	34	59	27
																									12													23						
					11														24	32				21	21																		
	10																																										
																			22	28	10		29								14					33		16			13	36	26	10
								11										11	30	32	32	36	31					12		22	13	10				48		25	19	16	16	50	39	46
																					10																						
																		15	20	16	20	22	17	10						12	11	11				13		16	13	12	18	19	23	11
			10	11	10	2,453	1,848		32			3,139	20	5,490	12						38					17							45	21			13					37	17,301	15
																		13			28							26	10								32		40			88	42	27
124	118	81	39	43	33				101	267	83				30	35	33	297	5,500	86	211	396	155	55,746	68,460	45,759	94	164	139				133	285	77		63		63		5,022	130	48	56
27	26	20	39	55	14	12		36		10	25	116	21	99	197		72	31	65	57	51	24	29	54			143	27	15	73	97	92	209	46	385	80	86	74	39	45	48	66	14	60
14	10			12						13	11																																	
																														14													
243	200	226	250	288	208	179	185	184	251	279	200	224	212	227		12				11				11			15	12			18		117	51	138	30	45	23	23	55		56	37	30
									11													24					11	37			25		140	104	127	10				31				
																																											12
																										12																	
										54	20								31			27		1,272	1,633	1,041								13							40			
																	10																										
										12												14												21									4,972	3,386
		91																										252												300			
186	126	130		11			11	17	240	309	216	215	174	196	19			10							203	276	152	99	121	162	10		19	27					27				156	93	133
	32	10			14	11	12	49	11									73	145	96	99	109	93	52	50	40	45	40	56	80	99	54				119	58	72	67	106	72	188	133	97
	12				22			34										109	138	130	225	278	198	118	165	98	12	39	35	45	74	54				121	24	79	91	126	118	184	157	111
																		96	36	103				19	25	21				130		34					69	104	22			46		
	21		44				26					54		91										26	20	39	16				27							24	56					24
																															11												
																																									40	
228	199	249				61	30	59	278	3,573	319	487	467	509	13	10	35	59				14	29	562	528	535	373	396	384	73	88	99	333	78	111	146	145	179	52	104	101	417	316	425
																										11		11															
																																							25				
21												24				17		16			12			53	30	10												38	12			18		
32	25		12									14		55	22				16					152	139	87	12				26			10					6,965			11	32	15,356
			25		10							13		14	103		38	35	18	14							28	14		26	172	16	43	21	127	16	23	14	16		15	28		
678	541	5,646	11,221	871	4,137	2,824	2,370	1,408	691	723	547	11,456	5,090	11,130	45,750	281	16,820	8,742	9,969	10,880	1,281	1,593	6,303	2,643	2,937	1,718	13,492	10,374	5,134	18,796	26,158	15,559	27,572	14,386	47,669	13,077	9,884	8,463	10,887	8,219	12,621	21,225	3,668	3,096
																				5,467																								
																			175	12	14	26	29								13					19		30		12		29		11
																		2,554	5,932	4,347		2,844	12	14				19	12		3,823	12		13			8,122		5,729				12	5,460
																		481	11,393		2,011	6,844	57				31	48			37										2,133		48	
25,527	209	26,971	81	17,409	26,733	30,599	15,112	16,164	116	225	94	257	9,312	2,343	284	77,350	249	102,802	155,535	117,282	110,352	83,953	62,439	29,372	31,732	23,386	51,150	12,938	32,255	15,823	36,704	35,607	306	53,691	183	95,514	293	32,831	154	68	4,159	120,173	400	63,629
						481	17	277		616	400						200	348									340	580	11	439	425	460	1,096	708	863	1,130	1,193	1,138	365	887	919		496	476
																								10																			29
																																											13,865
					36												14				24			275	428	322													149			141		
								11										35	57	60	60	80	35		35	41		12		36	58	36				73	11	55	29	41	52	98	63	59
			16	13	24		14	31	44	166	26					10	12	37			27	88		387	472	302		129	79				23	93	18		119	12	42		39	278	80	67
																		3,861	42	5,340	2,932	33	43	53	29	14		54											16				24	
																						21					14	28															
22	23	42	30	25	33	18	21	22	22	27	12	49	41	56	126		75	42	15	31			17				44	16	39	41	74	52	70	45	209	27		36	32	20	14	56		
																																				28						36	
	10	14			19			18										39	82	88	44	88	66	28	51	27	29	43	34	34	256	31				108	27	80	25	24	38	106	52	51
59	56	62	10	136	18	8,896	10,906		128	37			45	12			14	42	51	9,959	8,169	66	20,426	55	50	112	118	24,043	45	17,579	13	11	43	25		1,205	24,144					11	27	11,967
																		13			12	15		12	25	19		27	10								24		19			50	36	22
				12																																							
																										12		51	11					34		11	13						
																																							24				
545	485	5,358	163	117	131	64	68	73	132	485	135	50	128	64	311	240	274	443	21,627	18,545	28,699	6,702	13,877	62,736	74,933	50,584	209	231	245	162	30,230	6,887	290	741	238	121	133	154	66,694	67,199	38,548	43,998	63,496	43,199
			125	458		140	258		370				46							13			32					174		131						27	182							25
	23			11									16													22	31			15								81				
11		15					24					38												12			10																
28,213	2,834	41,829	12,344	19,687	31,883	47,174	37,835	21,063	2,719	6,974	2,168	17,423	16,287	20,525	47,449	94,644	20,489	176,610	252,742	215,959	171,874	109,787	127,234	166,987	197,772	134,934	121,924	115,760	111,363	107,404	117,644	93,730	36,418	70,851	67,610	133,487	101,008	62,687	106,045	90,440	65,797	213,018	108,537	166,928

Table S6: Taxonomic composition and read numbers of the 75 fish species from 90 libraries identified in HiSeq analyses of eDNA samples

Species number	Class	Order	Family	Species	A41	A42	A43	B41	B42	B43	C41	C42	C43	D41	D42	D43	E41	E42	E43	a41	a42	a43	b41	b42	b43	c41	c42	c43	d41
1	*Actinopterygii*	*Beloniformes*	*Hemiramphidae*	*Hyporhamphus intermedius*																15		24							
2	*Actinopterygii*	*Clupeiformes*	*Engraulidae*	*Coilia lindmani*																130	127	1,026	149	80	199	171	114	106	101
3	*Actinopterygii*	*Clupeiformes*	*Engraulidae*	*Coilia mystus*																	13	17	26	22	35	43	12	17	10
4	*Actinopterygii*	*Cypriniformes*	*Cobitidae*	*Cobitis elongatoides*																	16	22							
5	*Actinopterygii*	*Cypriniformes*	*Cobitidae*	*Cobitis lutheri*																									
6	*Actinopterygii*	*Cypriniformes*	*Cyprinidae*	*Acanthorhodeus chankaensis*	72	89	79	66	76	58	76	66,327	65	174	32,569	48	23	46	17	3,306	1,123	4,209	15	23	14	23,955	52		43
7	*Actinopterygii*	*Cypriniformes*	*Cyprinidae*	*Acheilognathus imberbis*		10	10											13	14					13	16				
8	*Actinopterygii*	*Cypriniformes*	*Cyprinidae*	*Acheilognathus meridianus*																									
9	*Actinopterygii*	*Cypriniformes*	*Cyprinidae*	*Acrossocheilus fasciatus*				117		263							301	434	588	215	176	131	2,656	224	666	561	82	168	120
10	*Actinopterygii*	*Cypriniformes*	*Cyprinidae*	*Acrossocheilus kreyenbergii*	1,583	61	59	72	88	151	22	102	33	75	192	48	842	15,495	5,666	8,242	11,056	13,018	91,267	41,678	62,613	25,806	30,193	32,462	253
11	*Actinopterygii*	*Cypriniformes*	*Cyprinidae*	*Acrossocheilus parallens*		42	38	163		341							520	712	11,581	243	247	165	1,567	309	791	810	102	711	189
12	*Actinopterygii*	*Cypriniformes*	*Cyprinidae*	*Ancherythroculter kurematsui*				81		117	118		24	134	83	19	93	43	66	101	81	103	273	100	44		31	34	
13	*Actinopterygii*	*Cypriniformes*	*Cyprinidae*	*Ancherythroculter lini*				13		24	50		10	53	21		19	13	28	19	25	30	57	30	10				
14	*Actinopterygii*	*Cypriniformes*	*Cyprinidae*	*Balantiocheilos melanopterus*																			35		373				
15	*Actinopterygii*	*Cypriniformes*	*Cyprinidae*	*Barbodes banksi*		37	23	104		266							262	483	634	172	129	98	916	178	432	413	64	125	89
16	*Actinopterygii*	*Cypriniformes*	*Cyprinidae*	*Barbodes binotatus*														34	87				61		45				
17	*Actinopterygii*	*Cypriniformes*	*Cyprinidae*	*Barbus ciscaucasicus*				40		83							100	151	261	71	59	35	548	74	173	156	15	51	41
18	*Actinopterygii*	*Cypriniformes*	*Cyprinidae*	*Carassius auratus*														10	186	13	13	12	85	27	65	17	31	38	
19	*Actinopterygii*	*Cypriniformes*	*Cyprinidae*	*Carassius carassius*																	13	10	57	68	45	25	20	19	
20	*Actinopterygii*	*Cypriniformes*	*Cyprinidae*	*Chanodichthys erythropterus*	28		18	53	13	118	169		48	219	84	33	93	51	77	84	2,471	92	266	102	46		31	26	
21	*Actinopterygii*	*Cypriniformes*	*Cyprinidae*	*Cyclocheilichthys janthochir*																									
22	*Actinopterygii*	*Cypriniformes*	*Cyprinidae*	*Cyprinus carpio 'mirror' x Cyprinus carpio 'singuonensis'*													11	30	43				87	12	41				
23	*Actinopterygii*	*Cypriniformes*	*Cyprinidae*	*Cyprinus megalophthalmus*													56	78	85				163	32	76				11
24	*Actinopterygii*	*Cypriniformes*	*Cyprinidae*	*Dawkinsia tambraparniei*																			10		11				
25	*Actinopterygii*	*Cypriniformes*	*Cyprinidae*	*Discherodontus ashmeadi*													23	24	30				89	26	28				
26	*Actinopterygii*	*Cypriniformes*	*Cyprinidae*	*Elopichthys bambusa*	27	29	22	27	21	18	46,320	816	22	23	45	23										13	29	12	
27	*Actinopterygii*	*Cypriniformes*	*Cyprinidae*	*Eremichthys acros*																									
28	*Actinopterygii*	*Cypriniformes*	*Cyprinidae*	*Gnathopogon strigatus*																									50
29	*Actinopterygii*	*Cypriniformes*	*Cyprinidae*	*Gobiobotia macrocephala*	60	51	42				103	339	98				437	496	438	11	2,544	10	49	109	53	392	450	289	412
30	*Actinopterygii*	*Cypriniformes*	*Cyprinidae*	*Hemibarbus labeo*													25	24	139										
31	*Actinopterygii*	*Cypriniformes*	*Cyprinidae*	*Hemibarbus umbrifer*													22	28	68										
32	*Actinopterygii*	*Cypriniformes*	*Cyprinidae*	*Hemiculter leucisculus*	23	123	75	299	420	389	181	150	46	174	216	19	160	43	69	97	89	92	258	168	40		19	25	
33	*Actinopterygii*	*Cypriniformes*	*Cyprinidae*	*Hemiculterella sauvagei*										45	25														
34	*Actinopterygii*	*Cypriniformes*	*Cyprinidae*	*Hypophthalmichthys molitrix*	21	10	12	226	272	123		73	89	446	949	214	156	268	239	39	25	43				51	14		
35	*Actinopterygii*	*Cypriniformes*	*Cyprinidae*	*Margariscus margarita*							12	47																	
36	*Actinopterygii*	*Cypriniformes*	*Cyprinidae*	*Onychostoma barbatulum*				57		97							135	225	304	75	74	38	399	94	234	203	22	57	49
37	*Actinopterygii*	*Cypriniformes*	*Cyprinidae*	*Osteobrama cotio*				30		80							110	140	196	77	148	52	503	104	238	177	31	55	43
38	*Actinopterygii*	*Cypriniformes*	*Cyprinidae*	*Platygobio gracilis*																									
39	*Actinopterygii*	*Cypriniformes*	*Cyprinidae*	*Procypris rabaudi*																									
40	*Actinopterygii*	*Cypriniformes*	*Cyprinidae*	*Pseudaspius leptocephalus*																									
41	*Actinopterygii*	*Cypriniformes*	*Cyprinidae*	*Pseudohemiculter dispar*				25		42	41		12	71	22		23	14	28	24	18	22	98	24	19				
42	*Actinopterygii*	*Cypriniformes*	*Cyprinidae*	*Pseudolaubuca engraulis*	5,399	327	2,206	15,396	2,218	30,985	39,574	527	10,417	43,421	21,019	6,065	23,145	12,403	22,425	20,748	17,291	20,717	69,052	28,203	7,968	281	5,996	6,609	173
43	*Actinopterygii*	*Cypriniformes*	*Cyprinidae*	*Ptychocheilus umpquae*																									
44	*Actinopterygii*	*Cypriniformes*	*Cyprinidae*	*Puntioplites waandersi*				10		10							27	41	37	24	11	11	118		60	32		319	
45	*Actinopterygii*	*Cypriniformes*	*Cyprinidae*	*Rutilus rutilus*																									
46	*Actinopterygii*	*Cypriniformes*	*Cyprinidae*	*Sarcocheilichthys lacustris*																									
47	*Actinopterygii*	*Cypriniformes*	*Cyprinidae*	*Sarcocheilichthys sinensis*																									
48	*Actinopterygii*	*Cypriniformes*	*Cyprinidae*	*Saurogobio immaculatus*																28		101							
49	*Actinopterygii*	*Cypriniformes*	*Cyprinidae*	*Schizothorax plagiostomus*				28		56							36	123	153	58	38	26	139	43	180	112	12	51	23
50	*Actinopterygii*	*Cypriniformes*	*Cyprinidae*	*Semotilus atromaculatus*	30	12	29				31	123	15	10		154	150	496	343		895	17				52	61	53	90
51	*Actinopterygii*	*Cypriniformes*	*Cyprinidae*	*Sinibrama macrops*				65	23	86	66		16	148	69	12	90	54	82	66	35	41	167	73	14		11	14	
52	*Actinopterygii*	*Cypriniformes*	*Cyprinidae*	*Spinibarbus denticulatus*				26		74							87	186	222	60	41	26	271	49	116	100	11	21	10
53	*Actinopterygii*	*Cypriniformes*	*Cyprinidae*	*Squalidus argentatus*	101	96	115	125,604	143	128	146	146	91	103	116	99	15	137	12,843	3,600	65	10				16	14	24	153
54	*Actinopterygii*	*Cypriniformes*	*Cyprinidae*	*Squalidus multimaculatus*													44	136	209										28
55	*Actinopterygii*	*Cypriniformes*	*Cyprinidae*	*Squalidus wolterstorffi*														48	19										
56	*Actinopterygii*	*Cypriniformes*	*Cyprinidae*	*Squalius lepidus*																									
57	*Actinopterygii*	*Cypriniformes*	*Cyprinidae*	*Tinca tinca*														323	362										
58	*Actinopterygii*	*Cypriniformes*	*Cyprinidae*	*Toxabramis houdemeri*										60	40														
59	*Actinopterygii*	*Cypriniformes*	*Cyprinidae*	*Xenocypris davidi*														199	109										
60	*Actinopterygii*	*Perciformes*	*Centrarchidae*	*Micropterus salmoides*													12	12	19							18	25		
61	*Actinopterygii*	*Perciformes*	*Gobiidae*	*Rhinogobius brunneus*																									
62	*Actinopterygii*	*Perciformes*	*Gobiidae*	*Rhinogobius cliffordpopei*																									
63	*Actinopterygii*	*Perciformes*	*Gobiidae*	*Rhinogobius giurinus*		95	22	133	46	108	77	119	44	2,417	157	137	32	582	61	26,129	20,264	39,030	66	142	64	257	36,485	146	168
64	*Actinopterygii*	*Salmoniformes*	*Salmonidae*	*Oncorhynchus formosanus*	596	889	471	423	390	208	1,025	1,304	541	726	520	336	66	69	172	45	70	48	91	38	35	458	192	212	78
65	*Actinopterygii*	*Salmoniformes*	*Salmonidae*	*Prosopium cylindraceum*	2,108	3,738	1,598	1,250	1,002	405	4,845	2,376	1,440	1,335	1,278	606	113	104	310	113	203	249	272	990	97	1,854	631	999	760
66	*Actinopterygii*	*Salmoniformes*	*Salmonidae*	*Salmo salar*		42	20	614	750	464	4,914	11	355	84	402					264	586	686				3,287	88	147	644
67	*Actinopterygii*	*Salmoniformes*	*Salmonidae*	*Salvelinus confluentus*							44	17																	
68	*Actinopterygii*	*Siluriformes*	*Bagridae*	*Coreobagrus brevicorpus*																41	75	68	666	135	223	413	163	162	73
69	*Actinopterygii*	*Siluriformes*	*Bagridae*	*Pelteobagrus eupogon*																125	183	150	1,780	270	527	954	416	338	191
70	*Actinopterygii*	*Siluriformes*	*Bagridae*	*Pelteobagrus intermedius*				269		35										68	6,275	86	1,448	370	601	703	270	457	367
71	*Actinopterygii*	*Siluriformes*	*Bagridae*	*Pseudobagrus ondon*	29	17											34	65	31	117	160	98	1,319	15,988	432	25,792	350	381	158
72	*Actinopterygii*	*Siluriformes*	*Bagridae*	*Pseudobagrus vachellii*				93		45							25	26	24	4,910	2,742	1,745	5,303	757	1,622	713	416	207	506
73	*Actinopterygii*	*Siluriformes*	*Bagridae*	*Tachysurus nitidus*	288	187	239	86	93	12,486	242	773	177	78	272	56	1,351	1,192	1,210	32,735	39,289	22,602	240,749	109,120	144,384	27,521	15,636	815	64,196
74	*Actinopterygii*	*Siluriformes*	*Siluridae*	*Silurus asotus*																33	56	48	11		11				24
75	*Actinopterygii*	*Siluriformes*	*Siluridae*	*Silurus lanzhouensis*																									

Note: (1) Site A, B, C, D and E are from Tian-e-Zhou Baiji National Nature Reserve; site a, b, c, d, e, f, g, h, i and j are from Poyang Lake. (2) Sample number A41, A42 and A43 represent 3 PCR replicates of the eDNA sample collected at sampling site A in spring, respectively; sample number A71, A72 and A73 represent 3 PCR replicates of the eDNA sa

d42	d43	e41	e42	e43	f41	f42	f43	g41	g42	g43	h41	h42	h43	i41	i42	i43	j41	j42	j43	A71	A72	A73	B71	B72	B73	C71	C72	C73	D71	D72	D73	E71	E72	E73
156	113	237	877	248	560	331	216	66	252	68	2,471	184	151	1,997	2,024	524	388	528	438	182	216	206	102	147	158	209	259	145	70	19	43	86	82	91
21	19	50	187	54	28	58	39				39	29	13	76	78	95	79	93	62	46	48	54	30	38	24	50	55	41	11		14	13	30	11
29	20	34	129	18	8,952	14,090	16,920	2,914	84	14	7,891	4,312	27	13,495	7,397	1,588	45	4,713	44	91	70	34				22	3,187	11				35	34	
					32	54	35							9,073	12,643	7,956	16,041	8,882	15,539	66	31	45												
					12	10																												
61	183	109	2,042	298	178	128	154	323	579	252	688	429	255	154	159	212	186	256	146		53	14				31	44	76						
13,526	335	201	119,801	7,679	13,058	14,116	9,960	26,643	51,683	28,216	2,028	11,511	6,068	5,273	9,336	4,869	2,369	4,996	5,133	101	131	2,447	47	30	54	1,010	3,231	2,019	27	80	23	987	500	51
105	265	176	2,499	639	254	173	201	375	766	6,036	1,165	540	341	230	257	265	271	366	175	10	72	46				48	67	251						
16	250				28	24	10		91	41					13	10							40		15							31	31	23
																																83		14
						21	12					10	18	11	10																			
68	167	77	1,279	264	150	102	121	213	440	257	500	349	181	129	133	154	168	210	131		66	34				40	46	94	58	10				
								10	24	15	36	12																						
19	52	37	596	157	76	47	56	84	144	64	235	141	76	59	43	72	70	75	53								10	32						
					23	18	18	28	50	23					14	960																		
					15		11	18	38	19																								
					28	20			43	38													46		19	16	13					40	26	38
			126	24				23	32	27	43	39	10																					
	24		201	40		17	34	31	68	31	76	50	27	25	15	13	26	43	29															
			101	20	11		13		35	26	40	33					14	23																
		38	16																				10	11	10	2,453	1,848					3,139	20	5,490
		13	89																															
49	350	53	635	174							76	74	99		21	24	29		30															
391	961	100,655	380,934	106,203	39,960	19,349	21,840				245	367	9,414	22,597	38,577	9,898	6,010	2,803	3,901	124	118	81	39	43	33				101	267	83			
30	84				40	33	10	24	108	84					22	34	13	39		27	26	20	39	55	14	12		36		10	25	116	21	99
																				14	10									13	11			
		29	73																	243	200	226	250	288	208	179	185	184	251	279	200	224	212	227
		496	2,697	423	246	211	177							286	451	102	46	34	38											54	20			
31	85	53	902	101	95	55	42	105	214	97	314	160	71	73	84	88	72	111	57		32	10				11	12	49						
37	67	28	658	141	94	226	67	126	197	131	335	202	97	116	106	139	153	156	94															
		11	36																															
						12	10																											
									13	10													25		10							13		14
155	20,176	185	18,483	218	4,374	5,331	696	393	11,670	6,312	1,771	15,443	182	232	4,643	889	215	192	911	678	541	5,646	11,221	871	4,137	2,824	2,370	1,408	691	723	547	11,456	5,090	11,130
		76	676																															
			117	24							52	24	14	11	11	12	23	32																
		33	223																															
						64	15																											
					918	5,852	2,040																											
		23	160			79	30				87	22	48	115	82																			
	37	13	552	30	47	13		59	136	61	166	80	31	17	30	45	11	36	38															
74	822	6,557	42,409	376	71	126	104				130	190	117	111	201	149	112	74	118				16	13	24		14	31	44	166	26			
11	48																			22	23	42	30	25	33	18	21	22	22	27	12	49	41	56
11	44	24	452	94	44	25	40	54	137	55	149	96	44	54	103	47	37	52	27		10	14												
81	12	46	32	21	38,531	22,079	12,211	50	22,479	13,697	68	60	46	123	6,069	51	59	3,655	61	59	56	62	10	136	18	8,896	10,906		128	37			45	12
28	341	95	972	107	21	13	21				76	43	65	18	20		25	13	18															
									22	10																								
		13	132		77	66			67	45													125	458		140	258							
																				11		15												
23	11	11	31,466	16																														
92	137	95	168	70	124	45,289	10,853	197	211	146	103	149	78	174	11,960	132	3,175	152	82	25,527	209	26,971	81	17,409	26,733	30,599	15,112	16,164	116	225	94	257	9,312	2,343
82	373	24	156	31	108	109	95	22	73	34	30	68	38		12	10	11	17	973	186	126	130					11	17	240	309	216	215	174	196
338	900	88	480	166	296	301	268	124	158	64	89	215	121	15	29	48	19	63	35	228	199	249				61	30	59	278	3,573	319	487	467	509
69		85		637											21	118										481	17	277		616	400			
66	144	132	65	128				314	825	269	120	136	139		28	32																		
128	280	318	119	556				791	1,969	583	298	378	325	44	82	69																		
103	288	253	53	229				463	1,376	375	242	224	225	27	52	48																54		91
152	238	266	111	260				653	1,627	482	332	308	262	30	57	54																		
202	1,147	929	398	1,162	180	7,258	45	1,704	5,264	1,211	1,139	1,718	660	87	124	146	40	66	10	32	25											14		55
32,517	57,579	147,149	801,326	176,255	78,434	61,997	58,456	75,338	219,570	57,323	98,129	122,390	31,012	38,469	42,173	47,819	31,057	43,720	52,906	545	485	5,358	163	117	131	64	68	73	132	485	135	50	128	64
30	33				27	6,322	1,878	123	256	2,345	14		1,552				11		10															

mple collected at sampling site A in summer, respectively; other sample numbers have similar meanings. (3) The Latin name of fish species and the higher level of classification are based on https://www.fishbase.se/.

a71	a72	a73	b71	b72	b73	c71	c72	c73	d71	d72	d73	e71	e72	e73	f71	f72	f73	g71	g72	g73	h71	h72	h73	i71	i72	i73	j71	j72	j73	Total read numbers
												11	37					140	104	127										458
43		39	350	270	286	623	624	516	432	700	384	88	41	73	77	113	155	71	42	171	73	84	33	379	334	349	89	372	198	23,056
			162	86	35	119	100	84	52	79	53	10		13	18	17	24	25	23	36	17	24		96	79	78	34	59	27	3,030
																														38
14	14																													28
19	21	18	2,550	2,966	35	22	104	24	3,733	4,007	2,964	22	5,630	20	9,030	22	3,603	18	68	17	22	6,044	13	29	15	16	7,773	31		267,561
			2,437	10	2,298	11	10	3,067	12	23	12																			78,353
																														22
			527	325	288	278	439	336	158	149	119	77	133	155	163	282	211				303	126	432	184	261	233	405	363	252	19,911
139	16,614	2,402	49,772	37,181	39,551	8,914	4,178	18,454	8,299	10,084	6,649	54,943	59,800	71,577	43,943	17,855	30,013	5,438	249	16,722	19,861	49,503	18,018	13,068	11,958	173	15,397	15,047	3,994	1,338,412
			251	356	389	7,234	702	400	208	203	229	94	127	159	232	317	202				434	143	314	304	347	227	825	491	352	48,664
153		70	30	49	60							83	32	11	83	86	138	268	34	194	43	68	69	47	60	47	61	25	27	3,906
43		20	19	15	12							11	10		12	39	29	31	15	56	13		11							835
			10		10				32	25	19	27	24	37	16	12	21				20	22								765
			113	344	199	178	216	221	120	337	107	61	145	154	119	196	129				287	108	172	156	170	262	380	265	219	14,524
						23	11	22																			27	16		423
			57	91	59	95	106	102	32	90	61	10	23	23	41	71	47				145	32	65	77	81	53	143	97	90	5,747
	22	11	39	24	29							55	59	75	35	13	24				77	41		11	11		10	23		2,190
			42	54	21	16		20				31	43	66	36	20	23				24	31								785
171		63	47	48	43							70	46	21	76	117	56	120	68	203	237	53	34	42	29	56	110	25	14	6,198
				24	32				21	21																				98
				22	28	10		29													33		16				36	26	10	758
			11	30	32	32	36	31							22	13	10				48		25	19	16	16	50	39	46	1,727
																														21
			15	20	16	20	22	17							12	11	11				13		16	13	12	18	19	23	11	805
																		45	21								37	17,301	15	77,901
																														102
													26	10													88	42	27	1,857
30	35	33	297	5,500	86	211	396	155	55,746	68,460	45,759	94	164	139				133	285	77				63		5,022	130	48	56	954,296
																														188
																														118
197		72	31	65	57	51	24	29				143	27	15	73	97	92	209	46	385	80	86	74	39	45	48	66	14	60	6,321
																														118
												15	12					117	51	138	30	45	23	23	55		56	37	30	7,360
									1,272	1,633	1,041																			9,286
			73	145	96	99	109	93	52	50	40	45	40	56	80	99	54				119	58	72	67	106	72	188	133	97	7,030
			109	138	130	225	278	198	118	165	98	12	39	35	45	74	54				121	24	79	91	126	118	184	157	111	7,883
																														47
																														22
												11		11																22
103		38	35	18	14							28	14		26	172	16	43	21	127	16	23	14	16		15				1,307
45,750	281	16,820	8,742	9,969	10,880	1,281	1,593	6,303	2,643	2,937	1,718	13,492	10,374	5,134	18,796	26,158	15,559	27,572	14,386	47,669	13,077	9,884	8,463	10,887	8,219	12,621	21,225	3,668	3,096	943,566
																														752
				175	12	14	26	29													19		30				29		11	1,365
																														256
																														79
																														8,810
									275	428	322																			1,800
			35	57	60	60	80	35		35	41				36	58	36				73	11	55	29	41	52	98	63	59	3,494
	10	12				27	88		387	472	302		129	79				23	93	18		119	12	42		39	278	80	67	56,913
126		75	42	15	31							44	16	39	41	74	52	70	45	209	27		36	32	20	14				2,642
			39	82	88	44	88	66	28	51	27	29	43	34	34	256	31				108	27	80	25	24	38	106	52	51	4,364
			42	51	9,959	8,169	66	20,426	55	50	112	118	24,043	45	17,579	13	11	43	25		1,205	24,144					11	27	11,967	401,722
						12	15		12	25	19		27	10													50	36	22	2,521
																														67
												12		51								11	13							119
																					27	182								2,275
												22	31																	153
																														334
																												4,972	3,386	39,971
			2,554	5,932	4,347		2,844	12					19	12		3,823	12											12	5,460	25,027
			481	11,393		2,011	6,844	57				31	48																	20,865
284	77,350	249	102,802	155,535	117,282	110,352	83,953	62,439	29,372	31,732	23,386	51,150	12,938	32,255	15,823	36,704	35,607	306	53,691	183	95,514	293	32,831	154	68	4,159	120,173	400	63,629	1,721,934
									203	276	152	99	121	162	10		19										156	93	133	14,513
13	10	35					14	29	562	528	535	373	396	384	73	88	99	333	78	111	146	145	179	52	104	101	417	316	425	44,498
												340	580	11	439	425	460	1,096	708	863	1,130	1,193	1,138	365	887	919		496	476	27,605
																														61
																														4,417
			96	36	103				19	25	21				130		34					69	104							11,511
									26	20	39																			15,137
									53	30	10																			49,896
									152	139	87																11	32	15,356	58,527
311	240	274	443	21,627	18,545	28,699	6,702	13,877	62,736	74,933	50,584	209	231	245	162	30,230	6,887	290	741	238	121	133	154	66,694	67,199	38,548	43,998	63,496	43,199	3,639,140
			3,861	42	5,340	2,932	33	43	53	29	14																			25,131
												14	28																	42

Table S7: Fish species identified across all samples within a region and season.

Samples	Number of species	Species
5 spring samples from Tian-e-Zhou Reserve	11	*Acanthorhodeus chankaensis, Acrossocheilus kreyenbergii, Chanodichthys erythropterus, Hemiculter leucisculus, Hypophthalmichthys molitrix, Oncorhynchus formosanus, Prosopium cylindraceum, Pseudolaubuca engraulis, Rhinogobius giurinus, Squalidus argentatus, Tachysurus nitidus*
5 summer samples from Tian-e-Zhou Reserve	10	*Acrossocheilus kreyenbergii, Coilia lindmani, Coilia mystus, Hemiculter leucisculus, Hypophthalmichthys molitrix, Pseudolaubuca engraulis, Rhinogobius giurinus, Sinibrama macrops, Squalidus argentatus, Tachysurus nitidus*
10 spring samples from Poyang Lake	17	*Acanthorhodeus chankaensis, Acrossocheilus fasciatus, Acrossocheilus kreyenbergii, Acrossocheilus parallens, Barbodes banksi, Barbus ciscaucasicus, Coilia lindmani, Oncorhynchus formosanus, Onychostoma barbatulum, Osteobrama cotio, Prosopium cylindraceum, Pseudobagrus vachellii, Pseudolaubuca engraulis, Rhinogobius giurinus, Schizothorax plagiostomus, Spinibarbus denticulatus, Tachysurus nitidus*
10 summer samples from Poyang Lake	6	*Acanthorhodeus chankaensis, Acrossocheilus kreyenbergii, Coilia lindmani, Pseudolaubuca engraulis, Rhinogobius giurinus, Tachysurus nitidus*

Supplementary figures

Figure S1: Rarefaction curves of fish species in the spring (**a** and **c**) and summer (**b** and **d**) for Tian-e-Zhou Baiji National Natural Reserve (T) and Poyang Lake (P).

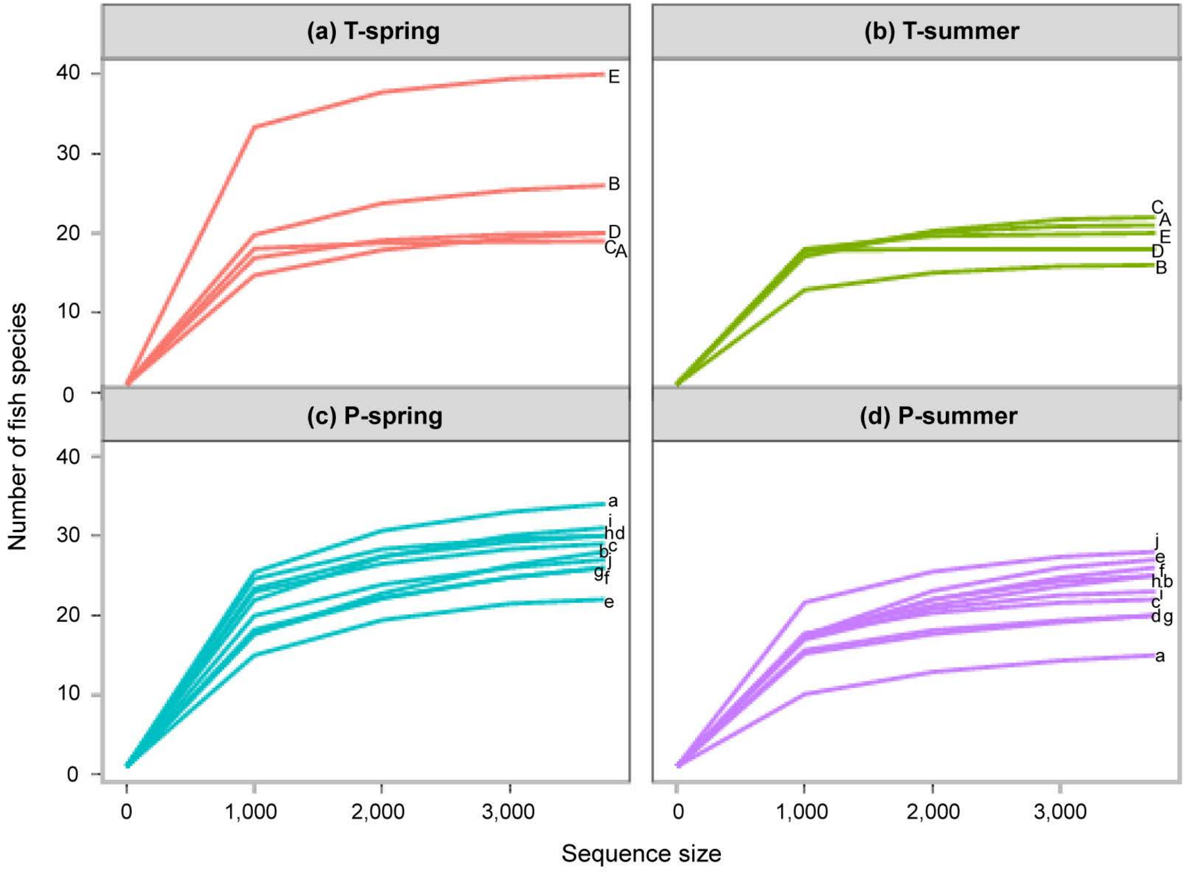

Figure S2: Distributions of fish species categorized into the type of depth they inhabit across sampling sites in the spring (**a** and **c)** and summer (**b** and **d**) for Tian-e-Zhou Baiji National Natural Reserve (T) and Poyang Lake (P).

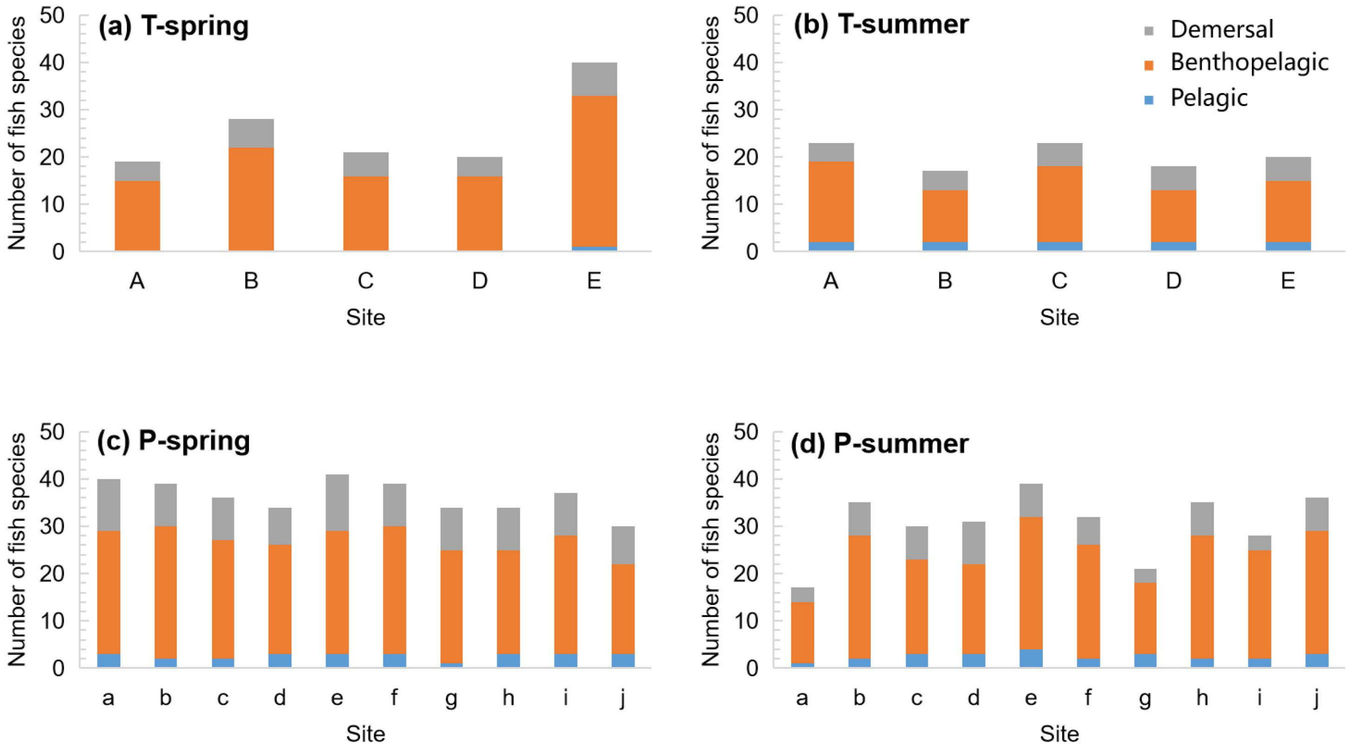

Figure S3: Distribution and relative proportion of fish species across regions and seasons.

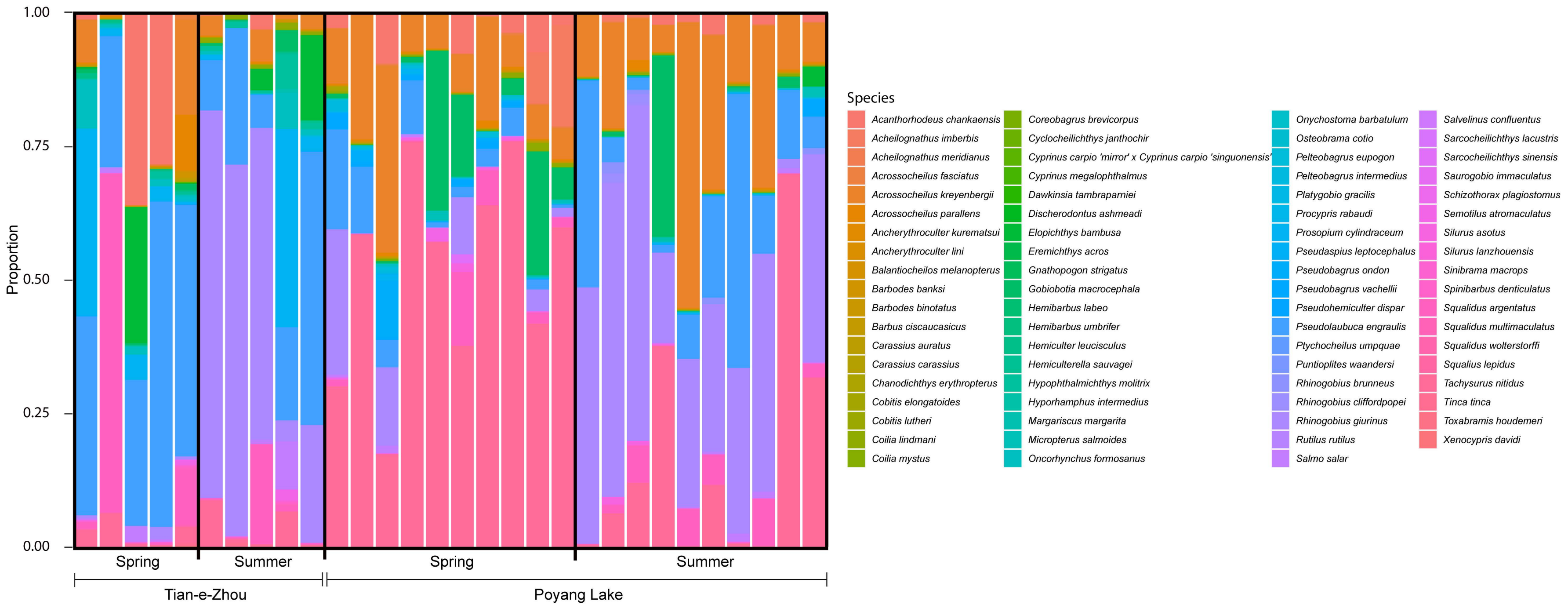

Figure S4: Distribution and relative proportion of fish families across regions and seasons.

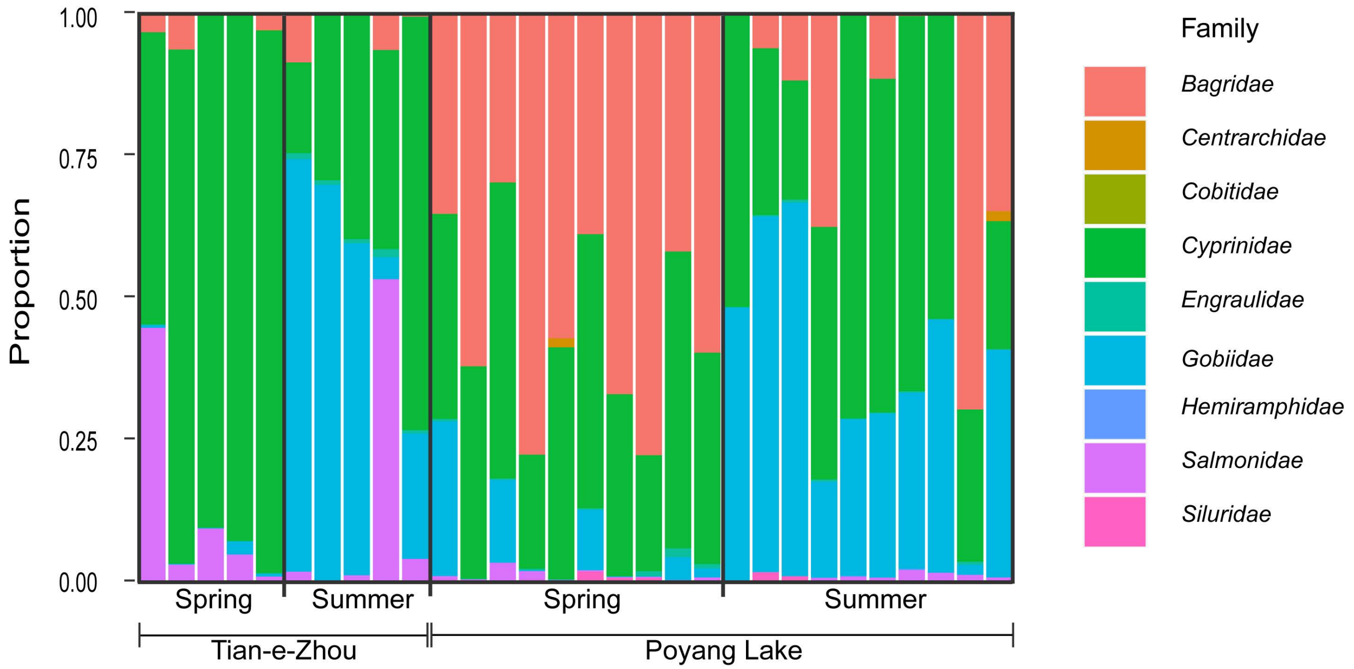

Supplementary literature cited

1. Yang, J. *et al.* A preliminary study on diet of the Yangtze finless porpoise using next-generation sequencing techniques. *Mar. Mammal Sci.* **35,** 1579-1586 (2019).

2. Hou, Y. Study on biology of finless porpoise from Yangtze river in captivity. *J. Aquacult.* **3,** 13-17 (1993). (In Chinese)

3. Chen, R., Wei, Y., Wu, L., Zheng, B. & Li, J. PAE coding system-based ethogram of Yangtze finless porpoise (*Neophocaena phocaenoides asiaeorientalis*) in a semi-natural environment. *Acta Theriol. Sinica* **35,** 40-47 (2015). (In Chinese)

4. Zhang, X. Reasons analysis and protection strategies of river dolphin risk in Tongling River Dolphin National Nature Reserve. *J. Tongling Univ.* **(1),** 102-105 (2017). (In Chinese)

5. Yu, D., Jiang, W. & Han, D. Observation on the adaptation of Yangtze finless porpoise (*Neophocaena phocaenoides asiaeorientalis*) to artificial feeding. *Acta Theriol. Sinica* **27,** 365-369 (2007). (In Chinese)

6. Zhu, Y., Liao, W., Feng, S. & Tan, H. Analysis of the Yangtze finless porpoise habitat characteristics. *Proceedings of the 4th National Conference on Hydraulics and Water Conservancy Informatics*, 140-146 (2009). (In Chinese)

7. Yu, D., Jiang, W. & Mi, L. Preliminary observations on feeding behavior of finless porpoises in a semi-nature reserve of Yangtze River. *Acta Theriol. Sinica* **23,** 198-202 (2003). (In Chinese)
